# Dual‐Activatable Nano‐Immunomodulator for NIR‐II Fluorescence Imaging‐Guided Precision Cancer Photodynamic Immunotherapy

**DOI:** 10.1002/advs.202409833

**Published:** 2024-10-14

**Authors:** Shanchao Diao, Zhifan Zhang, Sijun Zhao, Qiang Li, Xiaolong Zhang, Xiangqi Yang, Zhiwei Xu, Mingming Liu, Wen Zhou, Rutian Li, Chen Xie, Quli Fan

**Affiliations:** ^1^ State Key Laboratory of Organic Electronics and Information Displays & Institute of Advanced Materials (IAM) Nanjing University of Posts & Telecommunications 9 Wenyuan Road Nanjing 210023 China; ^2^ The Comprehensive Cancer Centre of Nanjing Drum Tower Hospital, the Affiliated Hospital of Medical School Nanjing University Nanjing 210008 China; ^3^ Clinical Cancer Institute of Nanjing University Nanjing 210008 China

**Keywords:** NIR‐II fluorescence imaging, photo‐activation, photodynamic therapy, photoimmunotherapy, phototheranostics

## Abstract

Photodynamic immunotherapy which combines photodynamic therapy with immunotherapy has become an important and effective method for the treatment of cancer. However, most cancer photodynamic immunotherapeutic systems are not able to achieve precise release of immunomodulators, resulting in systemic side effects and poor patient outcomes. Herein, a dual‐activatable nano‐immunomodulator (DIR NP), which both its photodynamic effect and agonist release can be activated under specific stimuli, is reported for precision cancer photodynamic immunotherapy. The DIR NP is self‐assembled from an R848‐conjugated amphiphilic polymer (mPEG‐TK‐R848) and a hydrophobic oxidized bovine serum albumin (BSA‐SOH)‐conjugatable photosensitizer (DIR). DIR NPs may generate a small amount of ^1^O_2_ under 808 nm laser irradiation, leading to the cleavage of thioketal (TK) moiety and release of R848 and DIR. The released DIR may conjugate with tumor‐overexpressed BSA‐SOH, improving its photodynamic efficiency and NIR‐II fluorescence signal. Such photodynamic efficiency improvement may further enhance the release of cargoes upon irradiation. The activated photodynamic effect induces immunogenic cell death (ICD) to release immune factors and R848 can enhance the maturation of dendritic cells for inhibiting the growth of both primary and distant tumors and eliminating lung metastasis. Therefore, this study provides a dual‐activatable intelligent nano‐immunomodulator for precise regulation of tumor photodynamic immunotherapy.

## Graphical abstract

A dual‐activatable nanoimmunomodulator (DIR NP) is reported for NIR‐II fluorescence imaging‐guided precision cancer photodynamic immunotherapy. DIR NP is prepared by encapsulating R848 with reactive oxygen species responsive amphiphilic copolymer mPEG‐TK‐R848. Upon 808 nm laser irradiation and oxidized bovine serum albumin treatment, the PDT efficiency, R848 release, and NIR‐II fluorescence signal can simultaneously be activated for photodynamic immunotherapy with high specificity.

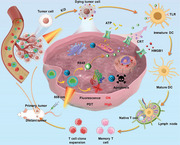

## 1 Introduction

In recent years, with the deepening understanding of immunology, photo‐immunotherapy which combines phototherapy and immunotherapy has attracted great attention as an emerging and powerful treatment method for tumors.^[^ ^1^, ^2^, ^3^ ^]^ The typical process of photo‐immunotherapy includes induction of immunogenic cell death (ICD) by phototherapy, maturation of dendritic cells (DCs), recruitment of effector T cells, and killing of both primary and distant tumors.^[^ ^4^, ^5^, ^6^ ^]^ Owing to the advantages of phototherapy such as high specificity and good therapeutic efficacy, phototherapy can induce efficient ICD without causing severe side effect.^[^ ^7^, ^8^, ^9^, ^10^ ^]^ Until now, a variety of materials have been designed for photo‐immunotherapy, which includes semiconducting polymers,^[^ ^11^ ^]^ small‐molecular dyes,^[^ ^12^ ^]^ covalent‐organic frameworks (COFs)^[^ ^13^ ^]^ as well as inorganic nanomaterials such as metal—organic frameworks (MOFs)^[^ ^14^ ^]^ and gold nanomaterials.^[^ ^15^ ^]^ These materials can also be cooperated with toll‐like receptor (TLR) agonists which can stimulate the maturation of DCs to form multifunctional nanosystems.^[^ ^16^, ^17^, ^18^ ^]^ Such nanosystems have demonstrated their excellent therapeutic efficacy and shown great potential in cancer therapy.

Although the great promise of nanosystem‐mediated photoimmunotherapy, the issue of potential side effect is need to be addressed.^[^ ^19^ ^]^ In most of photoimmunotherapeutic nanosystems, the two key components are photosensitizer and immune drug such as agonist.^[^ ^18^, ^20^ ^]^ Both photosensitizers and agonists are commonly loaded into nanoparticles via hydrophobic interaction. Such structure leads to the issue of burst release during circulation, which may cause undesirable distribution of cargos.^[^ ^21^ ^]^ The accumulation of agonist within normal tissue can result in severe whole‐body immune response and side effect.^[^ ^22^ ^]^ To improve the specificity, various studies have designed activatable agonists used for photoimmunotherapy.^[^ ^23^ ^]^ These agonists can only be activated or released upon the stimulation of exogenous light or tumor‐overexpressed endogenous stimuli which includes reactive oxygen species (ROS),^[^ ^24^ ^]^ GSH^[^ ^25^ ^]^ and enzymes,^[^ ^26^ ^]^ achieving a much better tumor specificity. For another key component (photosensitizer), most photoimmunotherapeutic nanosystems utilize “always‐on” photosensitizers as the phototherapeutic agents.^[^ ^27^ ^]^ Although photosensitizers only show photodynamic effect upon light irradiation, the accumulation in the normal tissue around the tumor may lead to the damage toward these tissue during phototherapy.^[^ ^28^ ^]^ Therefore, it is necessary to improve the formulation of nanosystems to further promote the specificity and reduce adverse effect of photoimmunotherapy.

**Figure 1:**
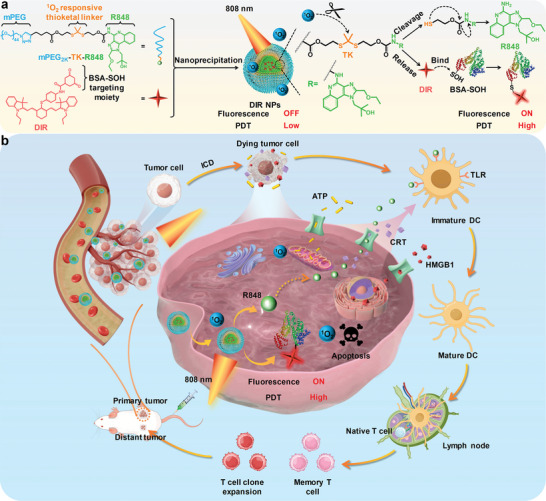
a) Schematic illustration of DIR NPs preparation and the mechanism of laser‐induced R848 release and conjugation of DIR with BSA‐SOH. b) Schematic illustration of photodynamic immunotherapy‐mediated by DIR NPs under 808 nm laser irradiation.

In this study, we report a dual‐activatable nano‐immunomodulator (DIR NP) for NIR‐II fluorescence imaging‐guided precision cancer photodynamic immunotherapy. DIR NPs are prepared by encapsulating DIR with mPEG_2k_‐TK‐R848 via nanoprecipitation. DIR is a 3,5‐dioxocyclohexanecarboxylic acid (DHCA)‐functionalized cyanine dye (IR780), while mPEG_2k_‐TK‐R848 is an amphiphilic prodrug which comprises hydrophilic poly(ethylene glycol) (PEG), ^1^O_2_ responsive thioketal (TK) linker and TLR agonist (R848). Because of the aggregation of DIR within nanoparticles, the NIR‐II fluorescence and PDT are greatly quenched. Upon 808 nm laser irradiation, DIR NPs produce small amount of ^1^O_2_, which cleaves part of TK linker to release R848 and DIR. It has been widely reported that the excessive expression of H_2_O_2_ in the cancer cells can reversibly convert thiols of bovine serum albumin (BSA) into sulfenic acids to form oxidized BSA (BSA‐SOH), and 3,5‐dioxocyclohexanecarboxylic acid (DHCA) is reported to specifically conjugate with sulfenic acid.^[^ ^29^, ^30^, ^31^, ^32^ ^]^ As DIR is modified with DHCA, the released DIR can further conjugate with tumor‐overexpressed BSA‐SOH, which allows its fluorescence recovery and greatly improves the ^1^O_2_ yield (**Figure** 1). The activation of ^1^O_2_ generation further leads to the acceleration dissociation of DIR NPs. The whole theranostic process of DIR NPs within tumor site is shown in Figure 1b. After accumulating into tumor site, DIR NPs are internalized into tumor cells. Both DIR and R848 are released upon 808 nm laser irradiation. The NIR‐II fluorescence signal and photodynamic effect of DIR are activated via the conjugation with BSA‐SOH. The activated DIR can mediate direct tumor ablation and ICD through photodynamic effect. Moreover, the released R848 can enhance the ICD‐stimulated immune response by promoting DC maturation through the activation of TLRs on the surface of DCs. The mature DCs will further recruit cytotoxic T cells to kill primary and distant tumors as well as inhibit lung metastasis. As such, DIR NP is a dual‐activatable nanoimmunomodulator, which both its TLR agonist and photosensitizer can be selectively activated within tumor site, for NIR‐II fluorescence imaging‐guided precise photoimmunotherapy.

## 2 Results and Discussion

### 2.1 Synthesis and Characterization

To prepare DIR NPs, mPEG_2k_‐TK‐R848 and DIR were synthesized (Scheme S1, Supporting Information). In order to synthesize mPEG_2k_‐TK‐R848, compound 1 with ^1^O_2_ responsive TK moiety was first synthesized by reacting acetone with thioglycolic acid. Then, the two carboxyl groups of compound 1 were reduced to hydroxyl groups by LiAlH_4_. The hydroxyl group on one side of compound 2 was transferred into alkynyl by conjugating with 5‐hexynoic acid via esterification. The other hydroxyl group was first reacted with 4‐nitrophenyl chloroformate and then substituted with resiquimod (R848) to give compound 5, and then its alkynyl group was reacted with azide‐capped PEG via click reaction to obtain the amphiphilic mPEG_2k_‐TK‐R848. To synthesize DIR that could conjugate with BSA‐SOH, 4‐aminothiophenol was first reacted with IR780 to modify the amino group onto IR780, then linked with DHCA via amidation. The successful synthesis of mPEG_2k_‐TK‐R848, DIR, and all the other intermediates were confirmed by proton nuclear magnetic resonance (^1^H NMR) and matrix assisted laser desorption ionization‐time of flight mass spectrometry (MALDI‐TOF MS) (Figures S1–S9, Supporting Information). The optical properties of DIR and IR780 were compared and studied. The absorption and emission spectra of DIR were slightly red‐shifted compared with those of IR780 in DMSO solution but showed no big difference (Figure S10, Supporting Information). Under 808 nm laser irradiation, both DIR and IR780 could generate ^1^O_2_ and superoxide anion (O_2_^·−^) (Figure S11, Supporting Information). These results showed that modification of DHCA had almost no influence on the optical properties of IR780.

**Figure 2:**
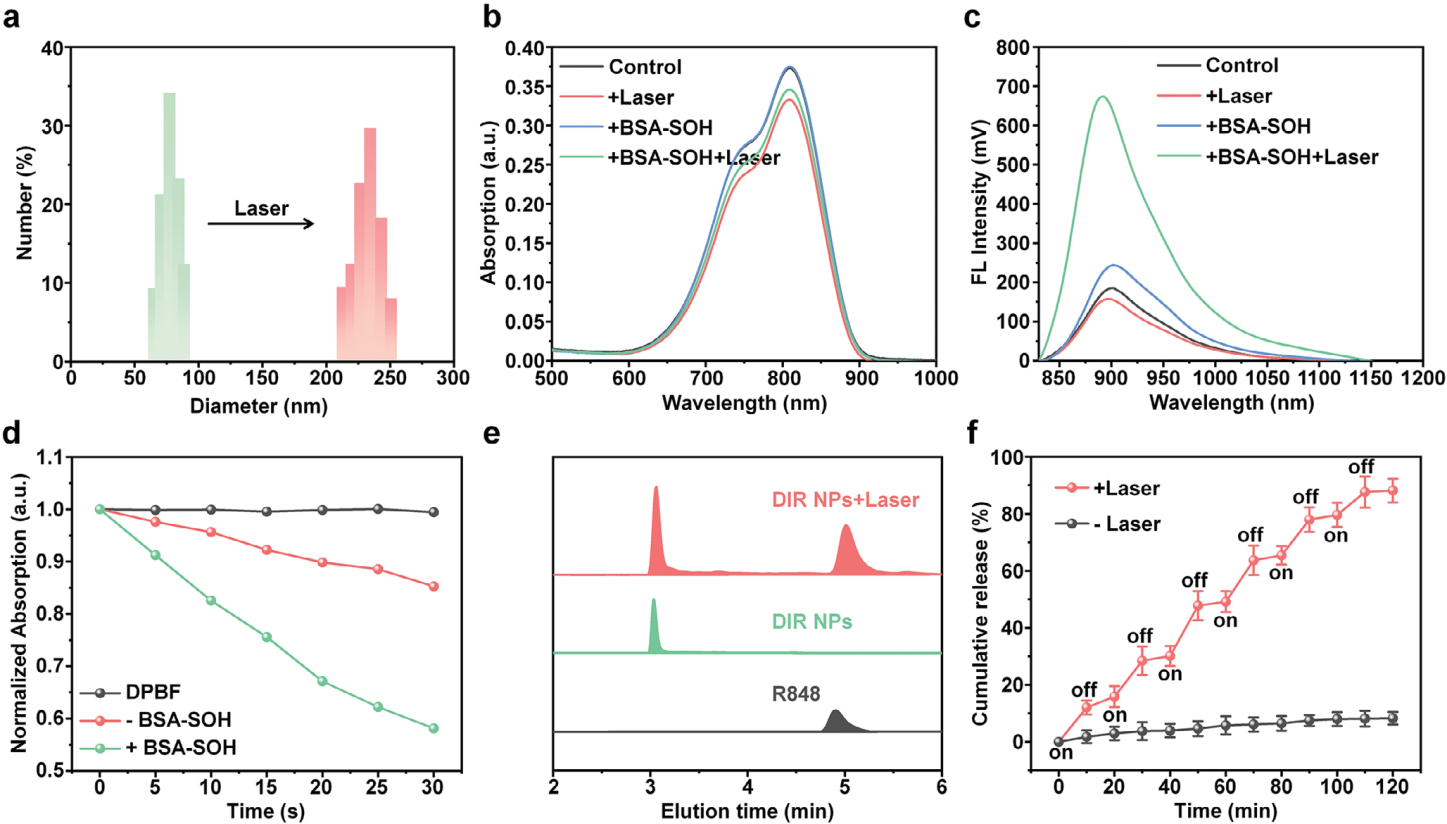
Characterization of DIR NPs. a) Hydrodynamic size distribution change of DIR NPs with or without 808 nm laser irradiation (0.3 W cm^−2^, 1 min) in PBS (pH = 7.4). b) Absorption and c) fluorescence spectra of DIR NPs (30 µg mL^−1^) with or without the 808 nm irradiation (0.3 W cm^−2^, 1 min) in PBS (pH = 7.4) or BSA‐SOH solution. d) Absorption changes of DIR NPs‐incubated DPBF at 414 nm with or without BSA‐SOH treatment under 808 nm photoirradiation (0.3 W cm^−2^) for different times. e) HPLC analysis of DIR NPs (30 µg mL^−1^) with or without 808 nm photoirradiation (0.3 W cm^−2^, 1 min). f) R848 release profile of DIR NPs (30 µg mL^−1^) in PBS (pH = 7.4) with or without laser irradiation. The laser wavelength was 808 nm with a power density of 0.3 W cm^−2^. The error bars represent standard deviations of three separate measurements (n = 3).

DIR NPs were prepared via a nanoprecipitation method by using mPEG_2k_‐TK‐R848 to encapsulate hydrophobic DIR. The loading efficiency for DIR was determined as 78.3%. As it shown in Figure S12 (Supporting Information), DIR NPs had a spherical morphology observed from transmission electron microscopy (TEM) images, and their diameter under dry state was in the range of 50–60 nm. The hydrodynamic size of DIR NPs was measured by dynamic light scattering (DLS), and the distribution was mainly in the range of 60–90 nm. The average size was calculated as 77.4 nm from DLS data (**Figure** **2**a, green bars), and the zeta potential of DIR NPs was determined as −7.37 ± 0.73 mV. The hydrodynamic size of DIR NPs remained essentially constant during 28 days of storage in PBS buffer or 5% FBS, indicating its good aqueous stability (Figure S13, Supporting Information). In contrast, the average hydrodynamic size of DIR NPs dramatically increased into 232.7 under 808 nm laser irradiation (Figure 2a, red bars). TEM images demonstrated that the regular spherical nanoparticles became irregular aggregates after laser irradiation (Figure S12, Supporting Information). As DIR NPs contained ^1^O_2_ responsive TK moiety, such result could be explained by the aggregation of DIR and R848 after cleavage of TK moiety by ^1^O_2_ generated from DIR. The optical properties of DIR NPs were then studied. DIR NPs showed a strong absorption peak at 808 nm with maximum emission at 900 nm and tailed to more than 1000 nm, showing the feasibility of NIR‐II fluorescence imaging. The mass extinction coefficient of DIR NPs was determined as 19.8 L g^−1^ cm^−1^ at 808 nm (Figure S14, Supporting Information), and the fluorescence quantum yield of aqueous solution of DIR NPs was calculated as 0.41% by using indocyanine green (ICG) as the reference (Figure S15, Supporting Information). Under 808 nm laser irradiation, the absorption showed slightly decrease, which could be attributed to the slightly aggregation of DIR after irradiation. In contrast, the absorption showed no obvious decrease in the presence of BSA‐SOH under irradiation, which showed that BSA‐SOH could prevent DIR from aggregation (Figure 2b). Under the same condition, the fluorescence intensity of DIR NPs significantly increased by 3.1‐fold compared with that without laser irradiation (Figure 2c). The possible explanation for this was that DIR may conjugate with BSA‐SOH via its 3,5‐dioxocyclohexane moiety. Such conjugation endowed DIR with good dispersity, thus enhancing its fluorescence signal.

The potential capability of cancer treatment for DIR NPs including photodynamic efficacy and drug release profile were then evaluated. DIR NPs showed a much better photostability than commercially available indocyanine green (ICG) under continuous laser irradiation for 10 min. Although the absorption of DIR NPs decreased slightly, the possible aggregation state change of DIR after irradiation made the fluorescence intensity remained stable (Figure S16, Supporting Information). 1,3‐Diphenylisobenzofuran (DPBF) was used to evaluate the ROS generation. DIR NPs could decrease the absorption of DPBF in some extent under 808 nm laser irradiation without BSA‐SOH, showing low photodynamic efficacy (Figure S17, Supporting Information). In contrast, the decrease rates were greatly improved in the presence of BSA‐SOH (Figure S18, Supporting Information). The correlation analysis indicated that under 808 nm laser irradiation (0.3 W cm^−2^) the ROS generation of DIR NPs increased 2.5‐fold by adding BSA‐SOH (Figure 2d). Such a result showed that the conjugation with BSA‐SOH may turn on the photodynamic efficacy of DIR. The release of R848 under different condition was then studied. After treatment with irradiation, a new elution peak at 5.1 min was observed for DIR NPs from high performance liquid chromatography (HPLC) analysis. Such elution time was consistent with the peak of R848, indicating that ^1^O_2_ generated from DIR could effectively cleave the TK moiety of mPEG_2k_‐TK‐R848 and release R848 (Figure 2e). With the increase of photoirradiation time, the release percentage of R848 increased gradually. Moreover, up to 85% R848 was released after 60 min of irradiation, showing an obvious photo‐activatable drug release manner. However, R848 was scarcely released from nanoparticles without photoirradiation (Figure 2f). Overall, these results further confirmed the destruction of nanoparticles by photoirradiation to achieve photo‐activation of drug release.

### 2.2 In Vitro Cell Studies

**Figure 3:**
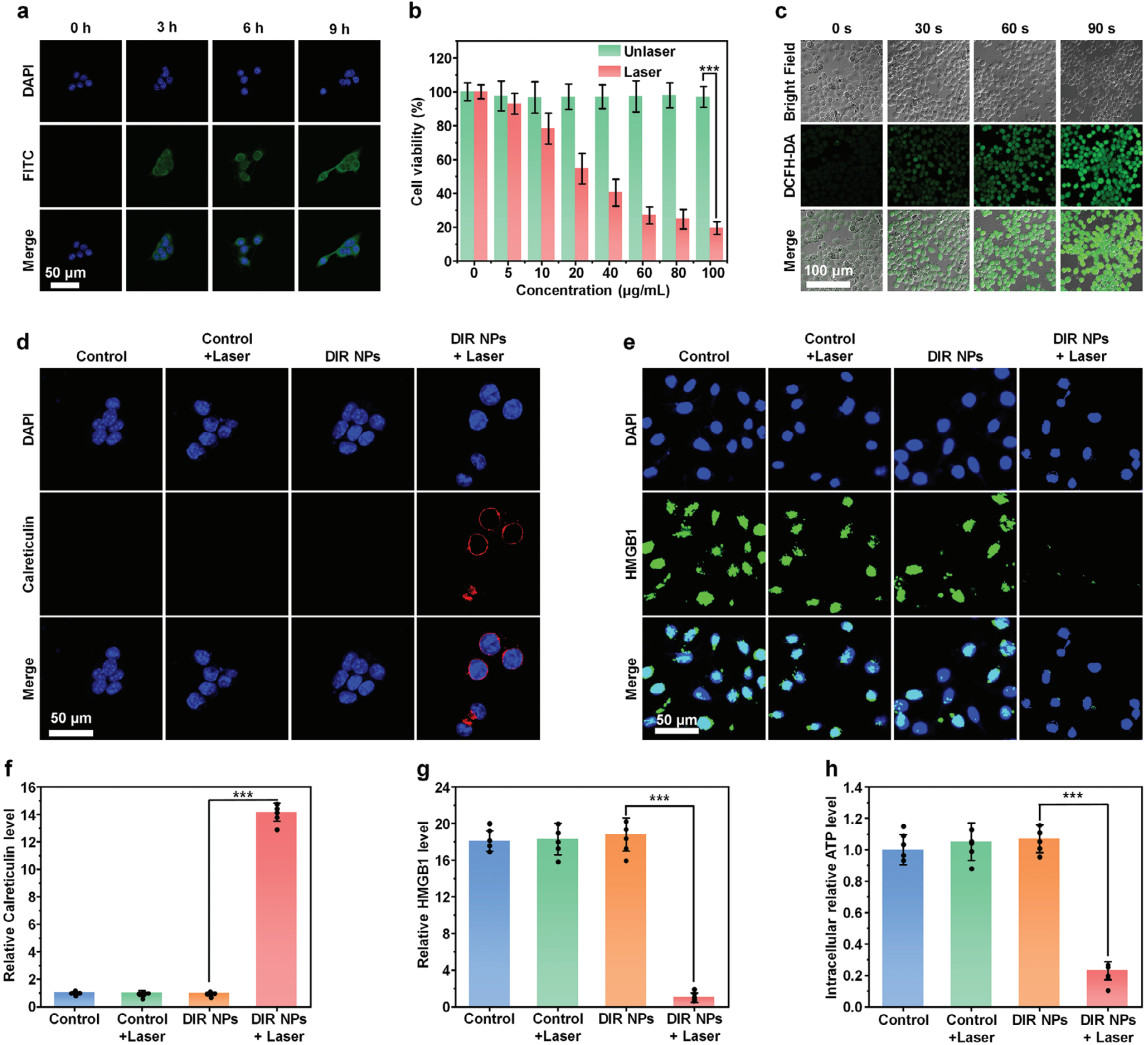
In vitro photodynamic effect and ICD induction. a) Confocal fluorescence images of 4T1 cells after incubation with DIR NPs for 0, 3, 6, and 9 h. The cells were co‐stained by 4′,6‐diamidine‐2′‐phenylindole dihydrochloride (DAPI, blue), and fluorescein isothiocyanate (FITC, green). The scale bars represent 50 µm. b) Viability of 4T1 cells treated with different concentrations of DIR NPs without or with 808 nm laser irradiation (0.3 W cm^−2^, 1 min). c) Fluorescence images of DCFH‐DA‐labeled 4T1 cells incubated with DIR NPs (20 µg mL^−1^) after different time of laser irradiation. The scale bars represent 100 µm. d) Fluorescence images and f) quantitative analysis of CRT exposure on the cell membrane surface after various treatments. The scale bars represent 50 µm. e) Fluorescence images and g) quantitative analysis of remnant HMGB1 in 4T1 cells after various treatments. The scale bars represent 50 µm. h) Intracellular ATP level in 4T1 cells after various treatments. The error bars represent the standard deviations of five separate measurements (n = 5). ^***^ *p* < 0.001.

The phagocytosis of DIR NPs by 4T1 murine mammary cancer cells was studied. As the fluorescence emission of DIR NPs was too long to be detected by confocal fluorescence microscopy. Fluorescein isothiocyanate (FITC) was encapsulated into DIR NPs to obtain F‐DIR NPs with strong green fluorescence. After incubating with F‐DIR NPs, evident green fluorescence signals could be observed within 4T1 cells (**Figure** **3**a). The maximum cellular uptake was observed at t = 9 h post‐incubation, and the results were also demonstrated in flow cytometry analysis (Figure S19, Supporting Information), verifying that F‐DIR NPs were effectively internalized by cancer cells through the endocytosis pathway. Next, in vitro anticancer activity of PDT was investigated. After 12 h incubating with different concentrations of DIR NPs, the 4T1 cells were treated with or without 808 nm laser irradiation and the cell viability was tested by MTT assay. Without laser irradiation, DIR NPs exhibited no cytotoxicity against 4T1 cells. However, the viability decreased gradually with the increase of DIR NPs concentration under 808 nm laser irradiation, and the viability was lower than 30% at the concentration of 100 µg mL^−1^ (Figure 3b). The IC_50_ of DIR NPs with laser irradiation was calculated as 23.8 µg mL^−1^, which demonstrated their outstanding photodynamic anticancer activity. Live/Dead assay and flow cytometry further demonstrated the effective in vitro anticancer efficacy of DIR NPs (Figure S20, Supporting Information). In addition, DIR NPs exhibited no cytotoxicity against NIH 3T3 cells even under the concentration of 100 µg mL^−1^, proving its good biocompatibility (Figure S21, Supporting Information). The intracellular ROS production levels were examined by using 2,7‐dichlorofluorescein diacetate (DCFH‐DA) as the fluorescent probe. As expected, DIR NPs‐incubated 4T1 cells under laser irradiation displayed an obvious green fluorescence signal recorded by confocal laser scanning microscopy (CLSM), and the signal increased significantly with the increase of irradiation time, demonstrating the ROS generation within cells (Figure 3c). Flow cytometry analysis further validated ROS generation under laser irradiation (Figure S22, Supporting Information). These results indicated the efficient ROS generation of DIR NP within cells and its good PDT efficacy against 4T1 cells.

The typical ICD process involved the release or expression of adenosine triphosphate (ATP), calreticulin (CRT), and high mobility group box 1 (HMGB1).^[^ ^33^ ^]^ To evaluate the capacity of DIR NPs for inducing ICD in cancer cells, the intracellular level of these biomarkers was measured. Confocal fluorescence imaging showed that obvious CRT release was detected only for the cells treated with DIR NPs and laser irradiation, and the CRT level was 14.1‐fold higher than that of DIR NPs without the laser group (Figure 3d,f). The small amount of CRT expression in cytoplasm could be attributed to the merge of cell membrane with cytoplasm during ICD.^[^ ^34^, ^35^ ^]^ HMGB1 released from nucleus during cell necrosis could bind to DC and activated the immune signaling pathway.^[^ ^36^ ^]^ Thus, determination of HMGB1 level within nucleus may indirectly characterize the release of HMGB1. As shown in the immunofluorescence staining images (Figure 3e–g), the 4T1 cells treated with DIR NPs showed very weak fluorescence intensity of HMGB1 in nuclei after laser irradiation compared with other groups, which indicated that HMGB1 was released from nuclei. The extracellular release of ATP played a vital role in promoting inflammation.^[^ ^37^ ^]^ The intracellular ATP level of DIR NPs with laser group was only 20% of other groups, indicating that DIR NPs with laser irradiation may promote the release of ATP from 4T1 cells (Figure 3h). These results demonstrated the occurrence of ICD induced by DIR NP‐mediated photodynamic effect.

**Figure 4:**
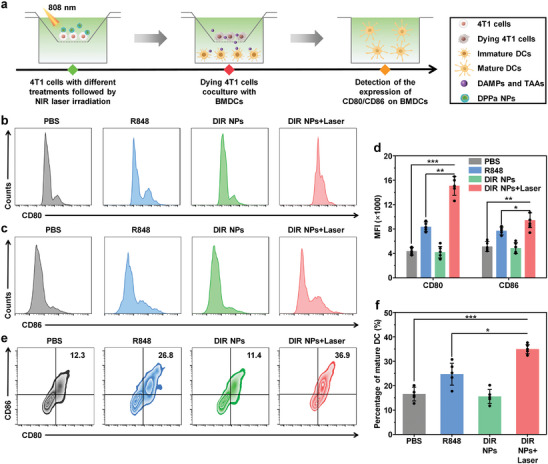
In vitro DC activation. a) Schematic illustration of BMDCs maturation induced by DIR NPs in vitro. Representative histograms of the cell surface expression of b) CD80 and c) CD86 on BMDCs under different treatments for 24 h. d) Quantitative analysis of mean fluorescence intensity (MFI) for CD80 and CD86 from flow cytometry results in (b) and (c). e) Representative flow cytometric plots of mature DCs (CD80^+^ CD86^+^ gated on CD11c^+^) under different treatments. f) Quantitative analysis of the amount of mature DCs from flow cytometry results in (e). [R848] = 5 µg mL^−1^, [DIR] = 20 µg mL^−1^, laser irradiation parameter (808 nm, 0.3 W cm^−2^, 5 min). The error bars represent standard deviations of five separate measurements (n = 5). ^*^ *p* < 0.05, ^**^ *p* < 0.01, ^***^ *p* < 0.001.

The maturation of DCs, which were the main antigen‐presenting cells, was crucial in introducing TAAs, activating T cells, and inducing immune response.^[^ ^38^ ^]^ The upregulation of co‐stimulating molecular CD80 and CD86 expression on the surface of DCs was considered as markers of the maturation of DCs.^[^ ^39^ ^]^ To study the in vitro DC maturation, 4T1 cells were seeded in the upper chamber with different treatments for 24 h, then immature bone marrow‐derived DCs (BMDCs) were placed in the bottom chamber and cultured for another 24 h, which was followed by examination of DC maturation using flow cytometry (**Figure** **4**a). Without laser irradiation, the expressions of both CD80 and CD86 from DIR NPs‐incubated 4T1 cells were almost the same with those of PBS‐incubated cells. Higher expressions were observed for R848‐incubated cells, showing that R848 may help the maturation of DCs. The highest expressions were detected in DIR NPs with laser group, which showed that DIR NP‐mediated PDT combined with R848 had the best efficiency on DC maturation (Figure 4b,c). The quantitative analysis indicated that the expression levels of CD80 and CD86 for DIR NPs with laser group were 3.1‐ and 2.3‐fold, respectively, higher than that of PBS group (Figure 4d). Based on these results, the percentage of mature DC after different treatments was studied. Similar with the results of CD80 and CD86 expression, DIR NPs with laser group had the highest percentage of mature DC (36.9%), which followed by the R848 group (26.8%), PBS group (12.3%) and DIR NPs group (11.4%) (Figure 4e,f). Consequently, these results demonstrated that DIR NP‐mediated photodynamic immunotherapy could greatly induce DC activation, and thus had a significant potential in augmenting antitumor responses.

### 2.3 In Vivo Tumor NIR‐II Fluorescence Imaging

**Figure 5:**
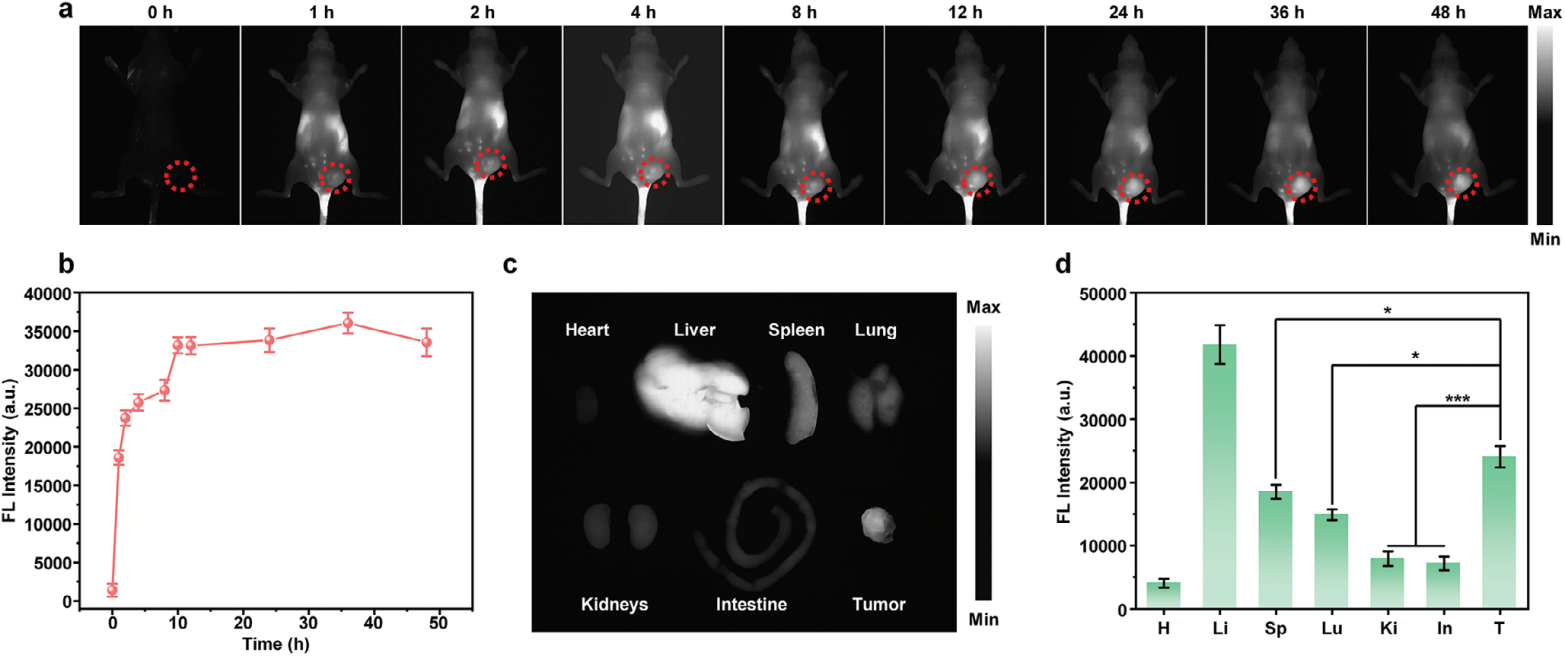
In vivo imaging. a) NIR‐II Fluorescence images of tumor‐bearing mice i.v. injected with DIR NPs (100 µL, 400 µg mL^−1^) at different time points. The red circles indicate the position of tumor. b) Fluorescence intensity of tumor area as a function of post‐injection time. Representative images c) and NIR‐II Fluorescence intensities d) of major organs and tumor (H = heart, Li = liver, Sp = spleen, Lu = lung, Ki = kidneys, In = intestine, T = tumor) resected from mice at 48 h post‐injection of DIR NPs. The error bars represent standard deviations of three different measurements (n = 3). ^*^ *p* < 0.05, ^***^ *p* < 0.001.

Owing to the intense NIR‐II fluorescence signal, DIR NPs were applied for in vivo NIR‐II fluorescence imaging of tumor. 4T1 tumor xenografted mice were used as the model. First, the penetration depth of NIR‐II fluorescence signal from DIR NPs in vitro was studied by using 1% intralipid (Figure S23, Supporting Information). It showed that the visibility of the capillaries deteriorated as the intralipid depth increased from 0 to 10 mm, and the penetration depth of NIR‐II fluorescence signal from DIR NPs was determined as 6 mm. Subsequently, the capability of NIR‐II fluorescence imaging of tumor for DIR NPs was evaluated in living mice. Before i.v. injection of DIR NPs, the mouse showed almost no NIR‐II fluorescence signal, which indicated the low tissue background signal in the NIR‐II region. At t = 1 h post‐injection, the fluorescence signal was monitored in the tumor of mice. Moreover, the fluorescence intensity of tumor regions gradually increased and reached the maximum at 36 h post‐injection, which was ≈28‐fold higher relative to the background (**Figure** **5**b). After 48 h post‐injection, mice were sacrificed and their major organs including tumor were collected for fluorescence imaging to evaluate the biodistribution of DIR NPs. Ex vivo biodistribution data showed that DIR NPs were mainly accumulated into liver (Figure 5c). Besides liver, tumor also exhibited strong NIR‐II fluorescence signal, showing the relatively high tumor accumulation of DIR NPs (Figure 5d). Meanwhile, the pharmacokinetic characteristics of DIR NPs was studied. The blood half‐life of DIR NPs was determined as 0.84 h (Figure S24, Supporting Information). These results demonstrated that DIR NP could effectively accumulate into tumor and light it up.

### 2.4 In Vivo Anticancer Therapy

**Figure 6:**
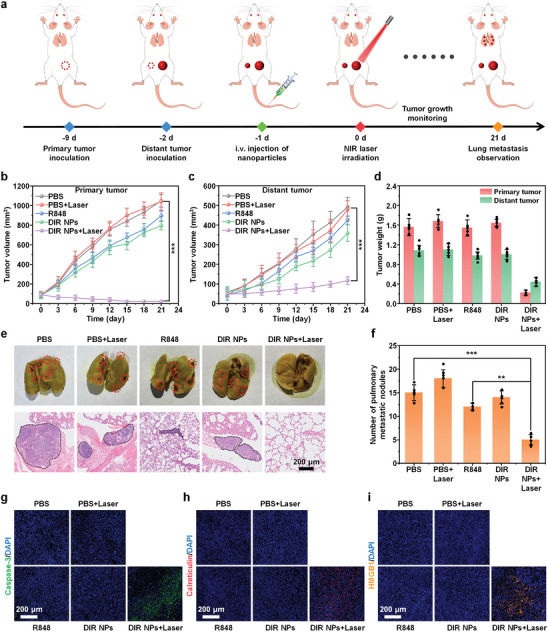
In vivo DIR NP‐mediated photodynamic immunotherapy. a) Schematic illustration of time schedule for tumor implantation, nanoparticle injection, cancer therapy, and monitoring of tumor growth and lung metastasis. Right side tumors were defined as primary tumors for laser irradiation, and left side tumors were defined as distant tumors without irradiation. b) Growth curves of primary tumors and c) distant tumors in 4T1 tumor‐bearing BALB/c mice following various treatments (n = 5). d) Weights of primary tumors and distant tumors following various treatments on day 21 (n = 5). e) Representative H&E staining of lung tissues from 4T1 tumor‐bearing BALB/c mice following various treatments on day 21. The scale bars represent 200 µm. f) Number of pulmonary metastatic nodules in mice following various treatments on day 21. The error bars represent standard deviations of five different measurements (n = 5). g–i) Immunofluorescence staining of caspase‐3 (green) (g), calreticulin (red) (h), and HMGB1 (yellow) (i) from primary tumor sections following various treatments on the third day. Nuclei were stained by DAPI (blue). The scale bars represent 200 µm. [R848] = 5 mg kg^−1^, [DIR] = 2 mg kg^−1^, 808 nm laser irradiation, 0.3 W cm^−2^, 10 min. ^**^ *p* < 0.01, ^***^ *p* < 0.001.

The in vivo anticancer efficacy of DIR NPs was then studied by using 4T1 xenografted mouse model. The tumor bearing mice were randomly divided into five groups which were PBS, PBS+Laser, R848, DIR NPs, DIR NPs+Laser. For the groups with laser irradiation, the irradiation was conducted at t = 24 h post‐injection. Tumor growth of primary and distant tumors was monitored every 3 day for 21 days (**Figure** **6**a). For the PBS group with or without laser irradiation, the tumor volume increased rapidly, indicating that PBS could not induce any photothermal or photodynamic effect under laser irradiation. Treatment groups with free R848 or DIR NPs without laser irradiation slightly delayed tumor growths relative to that in the PBS group, which may be attributed to the immunoactivation of R848. Remarkably, the DIR NPs+Laser group showed obvious inhibition against tumor growths of both primary and distant tumors, and the inhibition rates were calculated as 95.3% and 80.4% compared with PBS group, respectively (Figure 6b,c; Figure S25, Supporting Information). Similarly, the DIR NPs+Laser group outperformed other groups in the reduction of tumor weights in both primary and distant tumors (Figure 6d), the weights of primary and distant tumors were 0.22 and 0.42 g, which was 7.3‐ and 2.6‐fold lower than that in the PBS group, respectively. To further prove the efficacy of photodynamic immunotherapy, TUNEL staining of primary tumor in each group was carried out (Figure S26, Supporting Information). DIR NPs+Laser group showed obvious TUNEL expression, which was far higher relative to other groups, indicating that DIR NPs have excellent photodynamic immunotherapy effect. In addition, the hematoxylin and eosin (H&E) staining images revealed much severer cell apoptosis and necrosis in the primary and distant tumors for DIR NPs+Laser group than other groups (Figure S27, Supporting Information). Pulmonary metastasis after 21 days of treatments was examined to further explore the abscopal effects. Fewest pulmonary metastatic lesions were detected for the DIR NPs+Laser group among all the groups (Figure 6e), and the mean number of pulmonary metastasis nodules in the mice from DIR NPs+Laser group was 2.4‐ and 3.1‐fold fewer relative to that in the R848 group and the PBS group, respectively (Figure 6f). These results demonstrated that photodynamic immunotherapy mediated by DIR NP could not only kill primary tumor, but also inhibit the growth of distant and metastatic tumors maybe owing to the successful activation of whole‐body immune response. Moreover, the survival rate of mice in DIR NPs+Laser group remained 100% after 30 days of treatment, while the survival rate for other groups were less than 50% (Figure S28, Supporting Information). This confirmed the superior anticancer efficacy of DIR NPs with laser. For all the groups, the body weight of mice remained steady during treatments (Figure S29, Supporting Information), no histopathological damage was found in major organs (Figure S30, Supporting Information), and no notable elevation in biochemical parameters as evaluated by serum analysis (Figure S31, Supporting Information), demonstrating the good biosafety and biocompatibility of all the treatments. In a word, these data confirmed that DIR NP not only showed good anticancer efficacy via photodynamic immunotherapy, but also showed high biosafety during treatment.

To further evaluate the in vivo tumor cell apoptosis and ICD induction, primary tumors from mice on t = 3 day of treatments were resected for immunofluorescence staining. Caspase‐3 was a specific biomarker for cell apoptosis.^[^ ^40^ ^]^ The result showed that only DIR NPs+Laser group had obvious caspase‐3 expression, which was 24.6‐fold higher relative to the PBS group (Figure 6g; Figure S32, Supporting Information). The successful induction of in vivo ICD was characterized by the release or expression of ICD related biomarkers including CRT, HMGB1, and ATP. The immunofluorescence staining data demonstrated that only DIR NPs+Laser group could upregulate CRT and release HMGB1, which were 16.5‐ and 20.3‐fold higher than that in PBS group, respectively (Figure 6h,i; Figure S32, Supporting Information). Moreover, the levels of HMGB1 and tumor extracellular ATP were measured by enzyme‐linked immunosorbent assay (ELISA). The DIR NPs+Laser group significantly enhanced the release of HMGB1 and ATPs in primary tumor tissue (Figure S33, Supporting Information), which was 1.7‐ and 1.6‐fold higher relative to that in the PBS group, respectively. These results indicated that DIR NP‐based photodynamic therapy had the remarkable ability to induce in vivo cell apoptosis and ICD.

**Figure 7:**
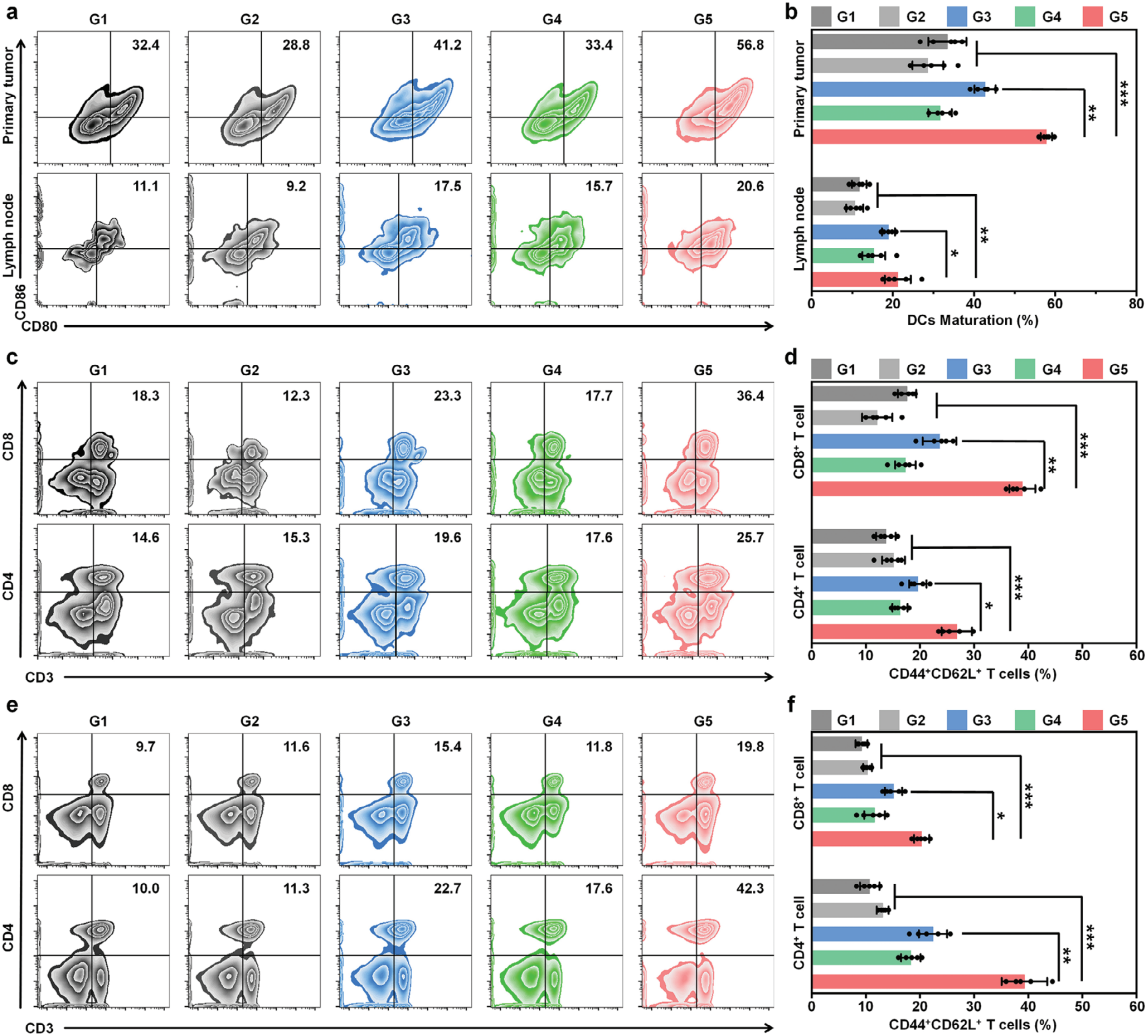
In vivo immune responses after NIR photodynamic immunotherapy. Representative flow cytometric plots a) and quantitative analysis b) of mature DCs (CD80^+^CD86^+^) in primary tumors and draining lymph nodes at day 3 after different treatments. Representative flow cytometric plots c) and quantitative analysis d) of central memory T cells (CD44^+^CD62L^+^) among CD4^+^ T cell subsets and CD8^+^ T cell subsets in primary tumors at day 10 after different treatments. Representative flow cytometric plots e) and quantitative analysis f) of central memory T cells (CD44^+^CD62L^+^) among CD4^+^ T cell subsets and CD8^+^ T cell subsets in tumor‐draining lymph nodes at day 10 after different treatments. G1, PBS; G2, PBS+Laser; G3, R848; G4, DIR NPs; G5, DIR NPs+Laser. The error bars represent standard deviations of five separate measurements (n = 5). ^*^ *p* < 0.05, ^**^ *p* < 0.01, ^***^ *p* < 0.001.

To explore the immune response mediated by DIR NPs, DC activation in primary tumors and tumor‐draining lymph nodes was evaluated by flow cytometry on day 3 after treatments. The DIR NPs+Laser group exhibited the highest expression of CD80 and CD86 (56.8%) among all the treatment groups, which was 1.7‐, 1.9‐, and 1.7‐fold higher than that of the PBS group (32.4%), PBS+Laser group (28.8%) and DIR NPs group (33.4%), respectively (**Figure** **7**a). More importantly, it was 1.4‐fold higher than that in the R848 group (41.2%). Such result indicated that the combination of PDT with TLR7 agonist (R848) had a better immune activation efficiency than single R848 (Figure 7b). Consistent with the results of DC activation in primary tumor, the proportion of mature DC in tumor‐draining lymph nodes for DIR NPs+Laser group was significantly higher than other groups, which may be attributed to the migration of mature DC from primary tumors (Figure 7a,b). Moreover, serum levels of proinflammatory cytokines (IL‐6; TNF‐α; and IFN‐γ) were detected at t = 3 day after different treatments by ELISA. The IL‐6, TNF‐α and IFN‐γ levels in DIR NPs+Laser group were 1.8‐, 3.1‐, and 2.6‐fold higher than that of the PBS group, respectively (Figure S34, Supporting Information), showing the good capability of immune activation by DIR NP‐mediated PDT.

Afterward, the activation of antitumor T cell response was evaluated by detecting the cytotoxic T lymphocytes (CD3^+^CD8^+^) and T helper cells (CD3^+^CD4^+^) in tumor tissues and tumor‐draining lymph nodes by flow cytometric analysis. For the primary tumor tissues, the highest populations of CD3^+^CD8^+^ and CD3^+^CD4^+^ T cells were observed in the DIR NPs+Laser group, which were 1.5‐ and 1.3‐fold higher than those in the R848 group, and 2.0‐ and 1.7‐fold higher than those in the PBS group, respectively (Figure 7c,d). In contrast, the number of cytotoxic T lymphocytes and helper T cells in the DIR NPs group without laser irradiation did not change significantly. Similar results were obtained for tumor‐draining lymph nodes. The levels of CD3^+^CD8^+^ and CD3^+^CD4^+^ T cell proliferation in the DIR NPs+Laser group were the highest among all the groups (Figure 7e,f). Furthermore, the effect of DIR NP‐mediated photodynamic immunotherapy was evaluated by determining the population of IFN γ^+^ T and GrzymeB^+^ T cells. The DIR NPs+Laser group showed 16% IFN γ^+^ T cells and 28.1% GrzymeB^+^ T cells among CD8^+^ T subsets in primary tumor after 10 days treatment, which were significantly greater than those of other groups (Figure S35, Supporting Information). In addition, similar results were obtained for tumor‐draining lymph nodes, the levels of IFN γ^+^ T cell and GrzymeB^+^ T cell proliferation in the DIR NPs+Laser group were highest among all the groups (Figure S36, Supporting Information). Consequently, all these results indicated that DIR NP‐mediated photodynamic immunotherapy could effectively stimulate the antitumor immune response and the infiltration of cytotoxic T cells in vivo.

## 3 Conclusion

In conclusion, we have reported a dual‐activatable nanoimmunomodulator (DIR NP) for NIR‐II fluorescence imaging‐guided precision cancer photodynamic immunotherapy. DIR NP is prepared by encapsulating BSA‐SOH‐targeting photosensitizer (DIR) with amphiphilic R848‐containing copolymer (mPEG‐TK‐R848). Under 808 nm laser irradiation, the TK moiety of DIR NPs was cleaved and lead to the slight release of DIR. The released DIR may conjugate with BSA‐SOH, leading to the enhancement of its ROS generation by 2.5‐fold and NIR‐II fluorescence intensity by 3.1‐fold. On the other hand, the enhanced ROS generation further lead to the acceleration release of cargoes, resulting in the release of up to 85% R848 within 120 min. Thus, the photodynamic effect and R848 release of DIR NP were activated by tumor overexpressed BSA‐SOH and laser irradiation, respectively. Such feature makes DIR NP a laser/BSA‐SOH dual‐activatable nanomedicine. The activated photodynamic effect could successfully induce 4T1 cell apoptosis and ICD. Combining with released R848, it could specifically trigger the maturation of DC in TLR‐expressing endosome, which played a crucial role in precision immunotherapy. DIR NP can effectively accumulate into tumor via passive targeting, and image the tumor through NIR‐II fluorescence imaging. Under 808 nm laser irradiation, DIR NP exhibited excellent tumor inhibition rate against primary tumor (95.3%). In addition, the DIR NP with laser irradiation was more effective on inducing DC activation in primary tumors (1.4‐fold) and tumor drainage lymph nodes (1.6‐fold) compared with native R848, eventually resulting in a higher infiltration of CD8^+^ T cells in tumors and whole‐body immune response for distant tumor (inhibition rate: 80.4%) and lung metastasis inhibition.

Our study thus designs a nanoplatform for dual‐activatable photoimmunotherapy. Besides R848, other immunomodulators or drugs can be used to conjugate onto amphiphilic copolymers to endow the nanoplatform with extended therapeutics. In addition, other kinds of photosensitizers instead of DIR can be encapsulated into nanoparticles to achieve better tumor specificity and therapeutic efficacy.

## Conflict of Interest

The authors declare no conflict of interest.

## Supporting information



Supporting Information

## Data Availability

The data that support the findings of this study are available from the corresponding author upon reasonable request.

## References

[1] V. Almeida‐Marrero , E. van de Winckel , E. Anaya‐Plaza , T. Torres , A. de la Escosura , Chem. Soc. Rev. 2018, 47, 7369. 30152500 10.1039/c7cs00554g

[2] Y. Zhou , Y. Zhang , C. Jiang , Y. Chen , F. Tong , X. Yang , Y. Wang , X. Xia , H. Gao , Small 2023, 19, 2300594. 10.1002/smll.202300594 36755191

[3] Z. Zhang , Z. Pan , Q. Li , Q. Huang , L. Shi , Y. Liu , Sci. Adv. 2024, 10, eadk0716. 38324678 10.1126/sciadv.adk0716 PMC10849581

[4] Z. Yang , Y. Teng , M. Lin , Y. Peng , Y. Du , Q. Sun , D. Gao , Q. Yuan , Y. Zhou , Y. Yang , J. Li , Y. Zhou , X. Li , X. Qi , ACS Nano 2024, 18, 7267. 38382065 10.1021/acsnano.3c13143

[5] X. Wang , G. Shi , R. Wei , M. Li , Q. Zhang , T. Zhang , C.‐F. Chen , H.‐Y. Hu , Chem. Sci. 2024, 15, 6421. 38699264 10.1039/d3sc06826a PMC11062115

[6] Z. Mai , J. Zhong , J. Zhang , G. Chen , Y. Tang , W. Ma , G. Li , Z. Feng , F. Li , X.‐J. Liang , Y. Yang , Z. Yu , ACS Nano 2023, 17, 1583. 10.1021/acsnano.2c11037 36595443

[7] X.‐F. Bai , Y. Chen , M.‐Z. Zou , C.‐X. Li , Y. Zhang , M.‐J. Li , S.‐X. Cheng , X.‐Z. Zhang , ACS Nano 2022, 16, 18555. 36341683 10.1021/acsnano.2c06871

[8] Y. Sun , R. Han , J. Wang , Y. Qin , Z. Ren , X. Feng , Q. Liu , X. Wang , J. Controlled Release 2022, 350, 734. 10.1016/j.jconrel.2022.08.057 36063959

[9] Q. Lin , Y. Wang , L. Wang , Z. Fan , Colloids Surf., B 2024, 235, 113770. 10.1016/j.colsurfb.2024.113770 38330689

[10] D. Xu , J. Ge , Y. An , S. Bai , Z. Wang , S. Wu , Q. Dai , Z. Lu , G. Liu , Small 2023, 19, 2300859. 10.1002/smll.202300859 37066745

[11] C. Zhang , J. Huang , M. Xu , J. Yu , W. Xin , S. He , K. Pu , Angew. Chem., Int. Ed. 2024, 63, e202405358. 10.1002/anie.202405358 38700137

[12] S.‐Z. Wang , Y. Guo , X. Zhang , H.‐H. Feng , S.‐Y. Wu , Y.‐X. Zhu , H.‐R. Jia , Q.‐Y. Duan , S.‐J. Hao , F.‐G. Wu , Adv. Funct. Mater. 2023, 33, 2303328.

[13] H. He , L. Du , H. Xue , J. Wu , X. Shuai , Acta Biomater. 2022, 149, 297. 35811069 10.1016/j.actbio.2022.07.003

[14] Z. Fan , H. Liu , Y. Xue , J. Lin , Y. Fu , Z. Xia , D. Pan , J. Zhang , K. Qiao , Z. Zhang , Y. Liao , Bioact. Mater. 2021, 6, 312. 32954050 10.1016/j.bioactmat.2020.08.005 PMC7475520

[15] Y. Feng , X. Ning , J. Wang , Z. Wen , F. Cao , Q. You , J. Zou , X. Zhou , T. Sun , J. Cao , X. Chen , Adv. Sci. 2023, 10, 2204842. 10.1002/advs.202204842 PMC9951300 36599677

[16] J. Li , N. Yu , D. Cui , J. Huang , Y. Luo , K. Pu , Angew. Chem., Int. Ed. 2023, 62, e202305200. 10.1002/anie.202305200 37194682

[17] M. He , M. Zhang , T. Xu , S. Xue , D. Li , Y. Zhao , F. Zhi , D. Ding , J. Controlled Release 2024, 368, 233. 10.1016/j.jconrel.2024.02.030 38395154

[18] Y. Chen , S. Zhi , J. Ou , J. Gao , L. Zheng , M. Huang , S. Du , L. Shi , Y. Tu , K. Cheng , ACS Nano 2023, 17, 16620. 37606341 10.1021/acsnano.3c02724

[19] J. Wu , K. Pu , Adv. Mater. 2024, 36, 2308924. 10.1002/adma.202308924 37864513

[20] B. Wang , D. Tang , J. Karges , M. Cui , H. Xiao , Adv. Funct. Mater. 2023, 33, 2214824.

[21] T. Su , X. Liu , S. Lin , F. Cheng , G. Zhu , Bioact. Mater. 2023, 26, 169. 36883121 10.1016/j.bioactmat.2023.02.016 PMC9982230

[22] J. Liu , S. He , Y. Luo , Y. Zhang , X. Du , C. Xu , K. Pu , J. Wang , Adv. Mater. 2022, 34, 2106654. 10.1002/adma.202106654 34854147

[23] G. Liu , J. Li , X. Wang , H. Ren , Y. Zhang , Adv. Healthcare Mater. 2024, 13, 2303305. 10.1002/adhm.202303305 38277491

[24] X. Han , D. Xiang , J. Li , S. Liao , D. Tang , Y. Han , M. Xu , W. Bi , H. Xiao , Nano Today 2024, 54, 102057.

[25] Q. Yang , G. Wu , Y. Yang , Y. Zhou , J. Song , H. Gao , Adv. Funct. Mater. 2024, 34, 2402194.

[26] A. Banstola , K. Poudel , J. O. Kim , J.‐H. Jeong , S. Yook , J. Controlled Release 2021, 337, 505. 10.1016/j.jconrel.2021.07.038 34314800

[27] C. Bhandari , M. Guirguis , N. A. Savan , N. Shrivastava , S. Oliveira , T. Hasan , G. Obaid , Nano Today 2021, 36, 101052. 33552231 10.1016/j.nantod.2020.101052 PMC7864390

[28] L. Xie , L. Wang , L. Li , C. Liu , L. Guo , Y. Liao , S. Zhou , W. Wu , Y. Duo , L. Shi , M. Yuan , ACS Appl. Mater. Interfaces 2024, 16, 5683. 38261396 10.1021/acsami.3c17977

[29] Y. H. Seo , K. S. Carroll , Proc. Natl. Acad. Sci. 2009, 106, 16163. 19805274 10.1073/pnas.0903015106 PMC2741475

[30] Y. Lyu , X. Zhen , Y. Miao , K. Pu , ACS Nano 2017, 11, 358. 27997794 10.1021/acsnano.6b05949

[31] L. J. Alcock , B. L. Oliveira , M. J. Deery , T. L. Pukala , M. V. Perkins , G. J. L. Bernardes , J. M. Chalker , ACS Chem. Biol. 2019, 14, 594. 30893551 10.1021/acschembio.8b01104

[32] Y. Gao , R. Sun , M. Zhao , J. Ding , A. Wang , S. Ye , Y. Zhang , Q. Mao , W. Xie , G. Ma , H. Shi , Anal. Chem. 2020, 92, 6977. 32314575 10.1021/acs.analchem.9b05855

[33] X. Liu , Y. Liu , X. Li , J. Huang , X. Guo , J. Zhang , Z. Luo , Y. Shi , M. Jiang , B. Qin , Y. Du , L. Luo , J. You , ACS Nano 2022, 16, 9240. 35713245 10.1021/acsnano.2c01669

[34] M. Ding , Y. Zhang , N. Yu , J. Zhou , L. Zhu , X. Wang , J. Li , Adv. Mater. 2023, 35, 2302508. 10.1002/adma.202302508 37165741

[35] C. Yang , M. Wang , M. Chang , M. Yuan , W. Zhang , J. Tan , B. Ding , P. a. Ma , J. Lin , J. Am. Chem. Soc. 2023, 145, 7205. 36958054 10.1021/jacs.2c12772

[36] Z. Liu , J. Zhang , H. Liu , H. Shen , N. Meng , X. Qi , K. Ding , J. Song , R. Fu , D. Ding , G. Feng , Adv. Mater. 2023, 35, 2208692. 10.1002/adma.202208692 36529696

[37] Q.‐X. Huang , J.‐L. Liang , M.‐T. Niu , X.‐K. Jin , C.‐Y. Dong , S.‐X. Cheng , X.‐Z. Zhang , Nano Today 2024, 56, 102253.

[38] X. Liu , R. Zhu , Y. Luo , S. Wang , Y. Zhao , Z. Qiu , Y. Zhang , X. Liu , X. Yao , X. Li , W. Li , Immunity 2021, 54, 2305. 34508661 10.1016/j.immuni.2021.08.012

[39] J. Liu , G. Liu , C. Dai , J. Wu , Q. Li , Chem. Eng. J. 2024, 485, 149446.

[40] B. A. Carneiro , W. S. El‐Deiry , Nat. Rev. Clin. Oncol. 2020, 17, 395. 32203277 10.1038/s41571-020-0341-y PMC8211386

