# Supplementary material for: Dual‐Activatable Nano‐Immunomodulator for NIR‐II Fluorescence Imaging‐Guided Precision Cancer Photodynamic Immunotherapy
Source: Adv Sci (Weinh). 2024 Oct 14;11(45):2409833. doi: 10.1002/advs.202409833 (PMC11615741; doi:10.1002/advs.202409833)
Supplement: Supplementary file 1 — Supporting Information [file ADVS-11-2409833-s001.pdf]

ADVANCED SCIENCE Open Access

Supporting Information

for *Adv. Sci.*, DOI 10.1002/advs.202409833

Dual-Activatable Nano-Immunomodulator for NIR-II Fluorescence Imaging-Guided Precision Cancer Photodynamic Immunotherapy

Shanchao Diao, Zhifan Zhang, Sijun Zhao, Qiang Li, Xiaolong Zhang, Xiangqi Yang, Zhiwei Xu, Mingming Liu, Wen Zhou, Rutian Li, Chen Xie* and Quli Fan**

Supporting Information

Dual-Activatable Nano-Immunomodulator for NIR-II Fluorescence Imaging-guided Precision Cancer Photodynamic Immunotherapy

Shanchao Diao[#], Zhifan Zhang[#], Sijun Zhao, Qiang Li, Xiaolong Zhang, Xiangqi Yang, Zhiwei Xu, Mingming Liu, Wen Zhou, Rutian Li, Chen Xie,* and Quli Fan**

1. Supporting figures

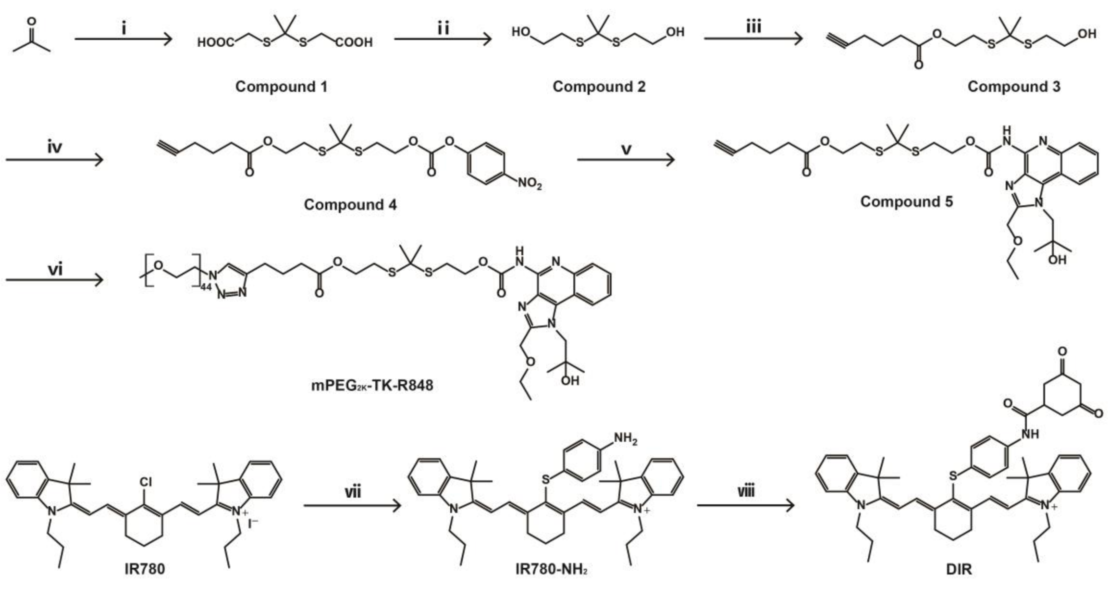

Scheme S1. Synthetic routes of mPEG$_{2k}$-TK-R848 and DIR. Reagents and conditions: (i) thioglycolic acid, dichloromethane (DCM), trifluoroacetic acid (TFA), room temperature (RT), 24 h; (ii) $LiAlH_4$ in tetrahydrofuran (THF), anhydrous THF, 0 °C, 2 h; (iii) 5-hexynoic acid, N,N'-diisopropylcarbodiimide (DIC), 4-dimethylaminopyridine (DMAP), anhydrous THF, RT, 24 h; (iv) 4-nitrobenzoyl chloride, triethylamine (TEA), anhydrous DCM, 0 °C, 1 h; (v) resiquimod (R848), N, N-diisopropylethylamine (DIPEA), 60 °C, 12 h; (vi) mPEG$_{2k}$-N_3, N N N' N' N''-pentamethyldiethylenetriamine (PMDETA), CuBr, anhydrous THF, N_2, RT, 24 h; (vii) 4-aminothiophenol, anhydrous DCM, RT, 24 h; (viii) 3,5-dioxocyclohexanecarboxylic acid (DHCA), 2-(7-azabenzotriazol-1-yl)-N,N,N',N'-tetramethyluronium hexafluorophosphate (HATU), 1-hydroxybenzotriazole (HOBT), DIPEA, anhydrous N,N-dimethylformamide (DMF), RT, 24 h.

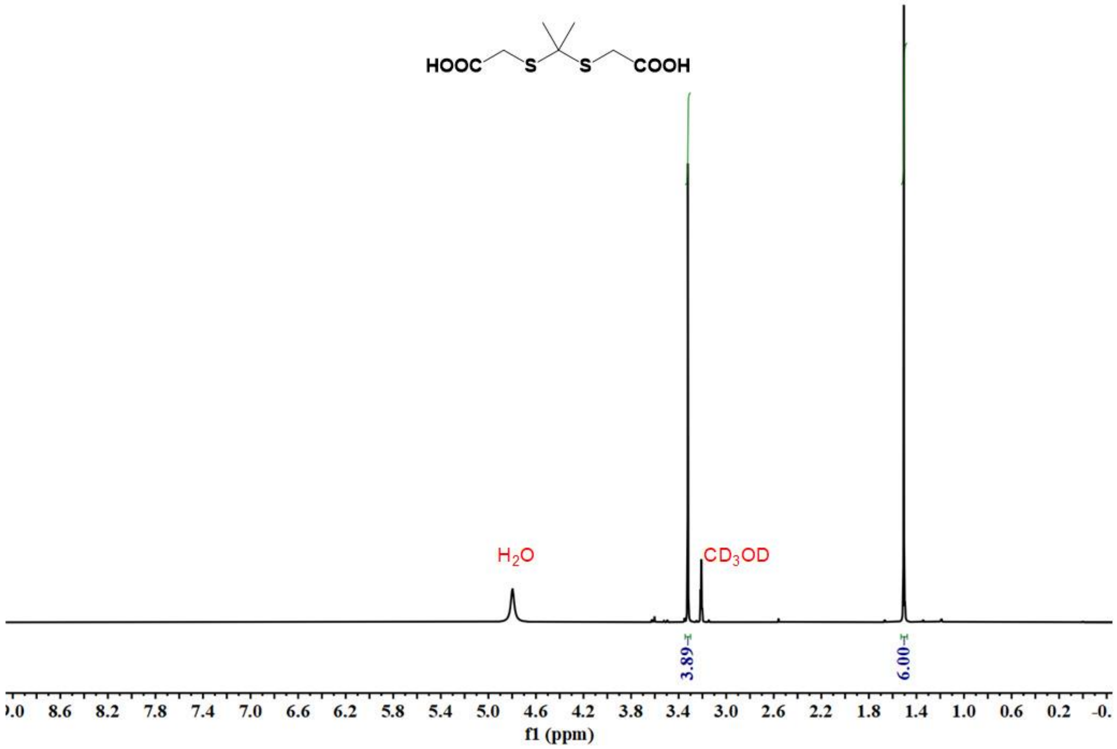

Figure S1. ^{1}H NMR spectrum of Compound 1. MeOH-d4 was used as solvent.

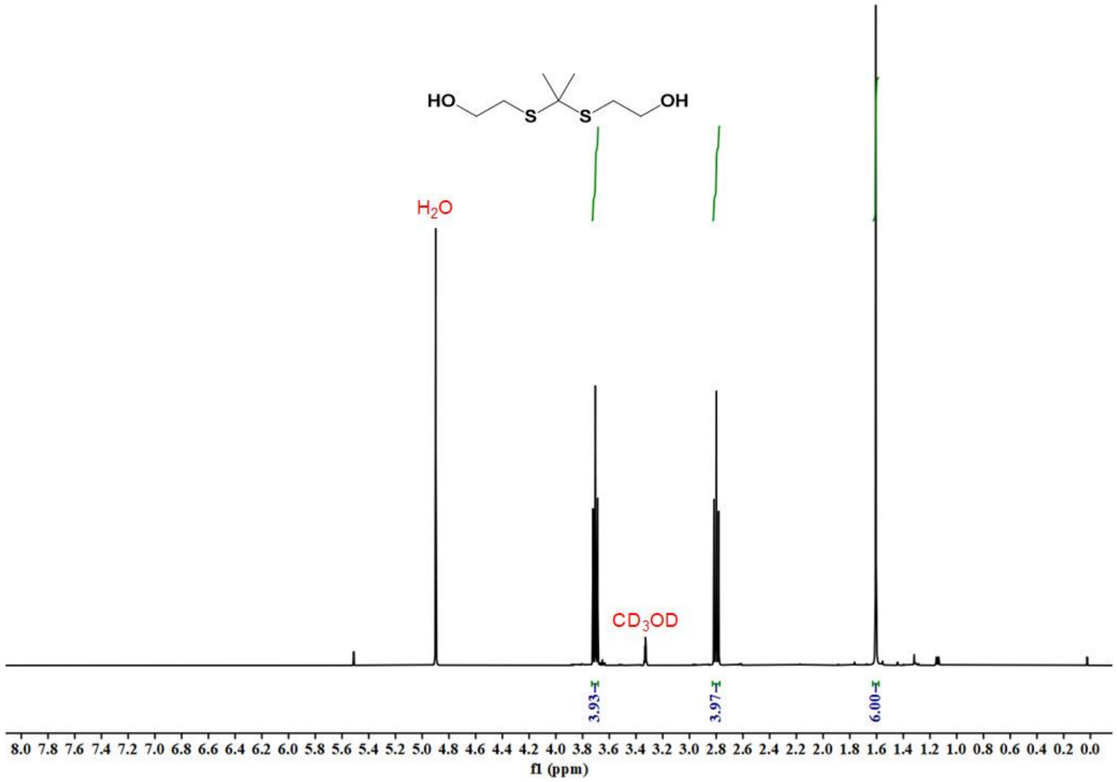

Figure S2. ^{1}H NMR spectrum of Compound 2. MeOH-d4 was used as solvent.

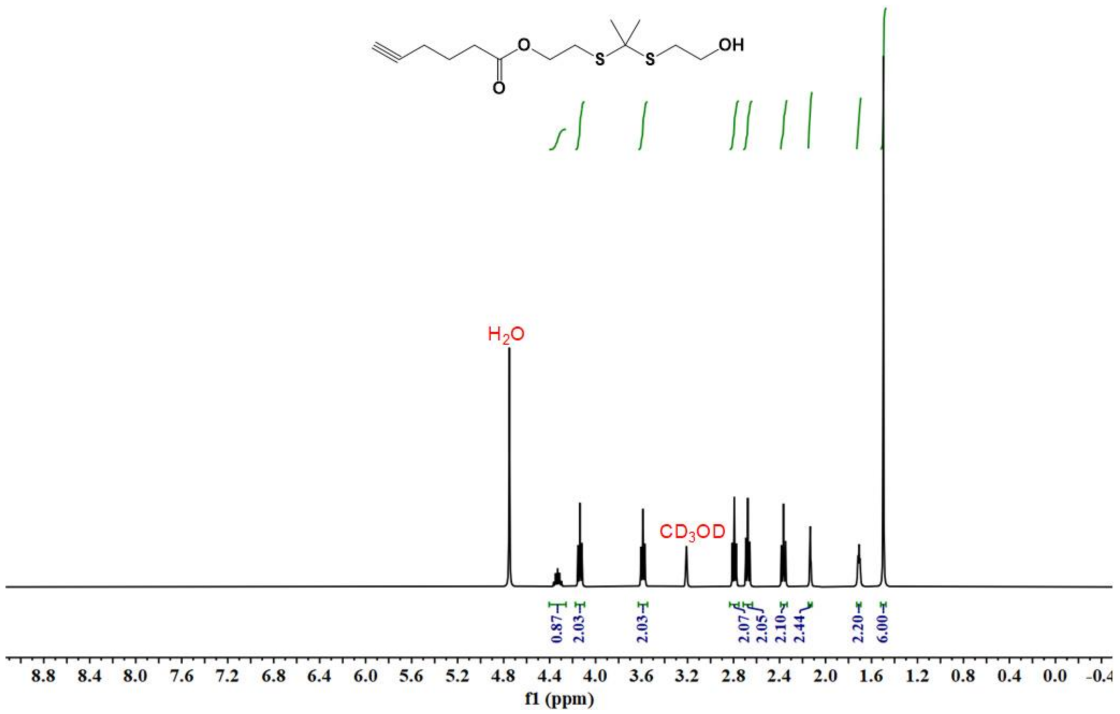

Figure S3. ^{1}H NMR spectrum of Compound 3. MeOH-d4 was used as solvent.

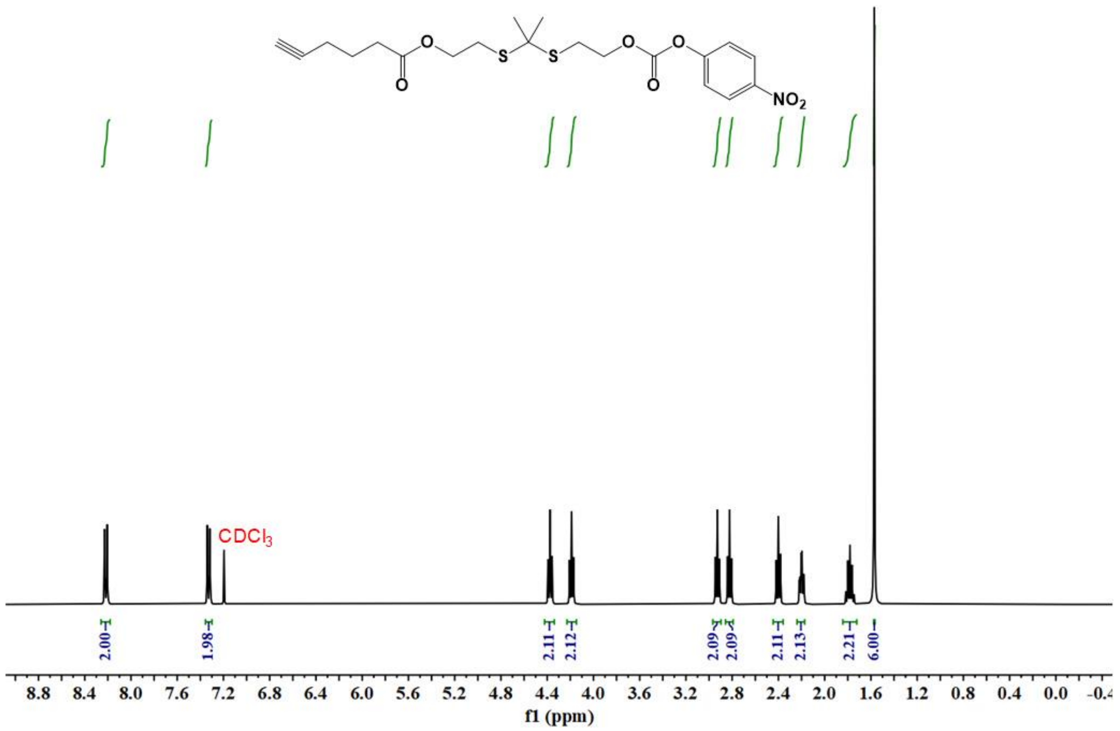

Figure S4. ^{1}H NMR spectrum of Compound 4. Chloroform-d was used as solvent.

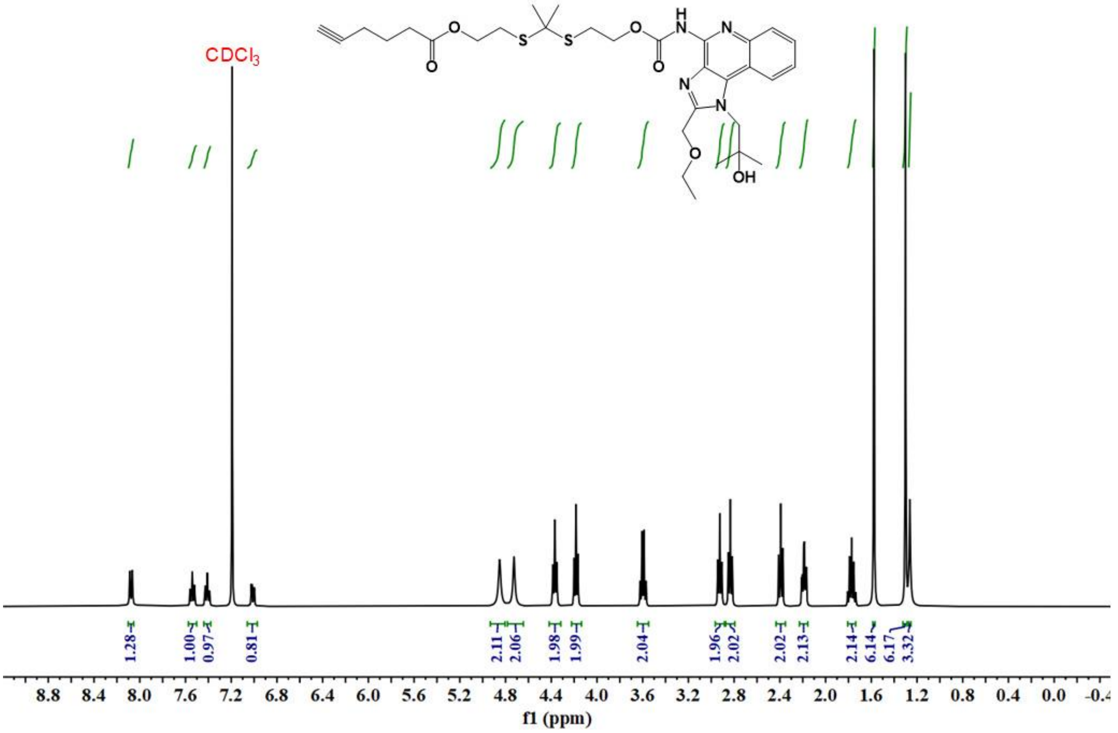

Figure S5. ^{1}H NMR spectrum of Compound 5. Chloroform-d was used as solvent.

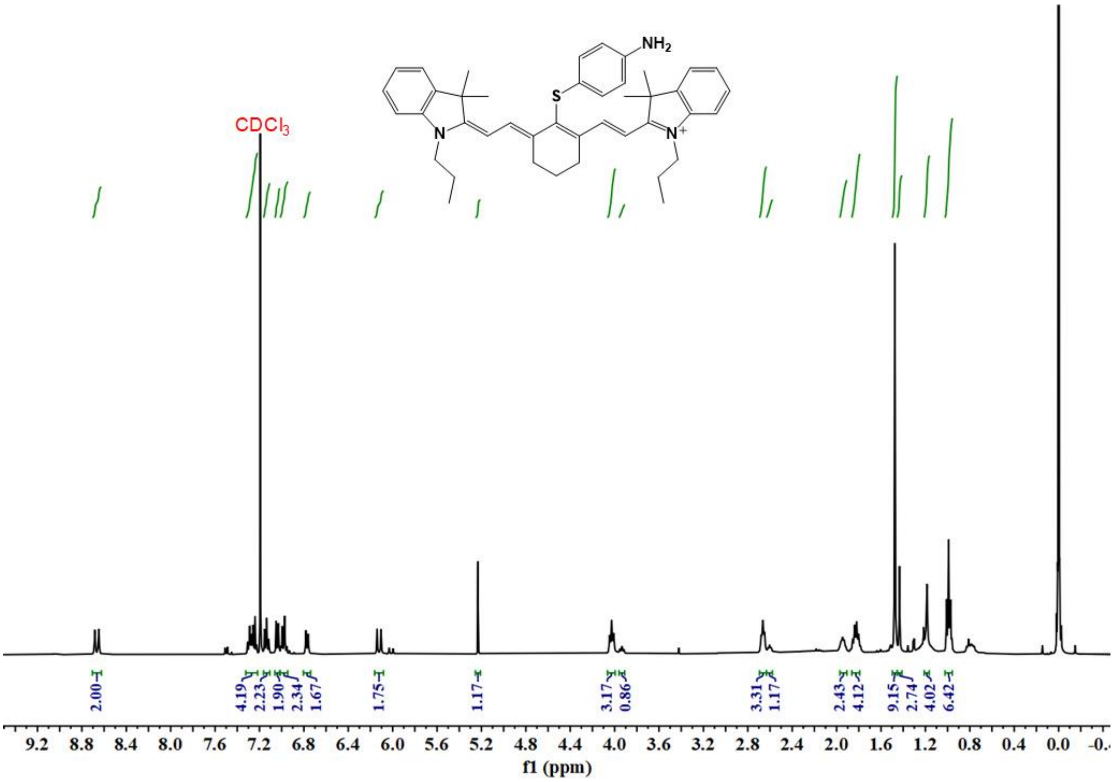

Figure S6. ^{1}H NMR spectrum of IR780-NH$_2$. Chloroform-d was used as solvent.

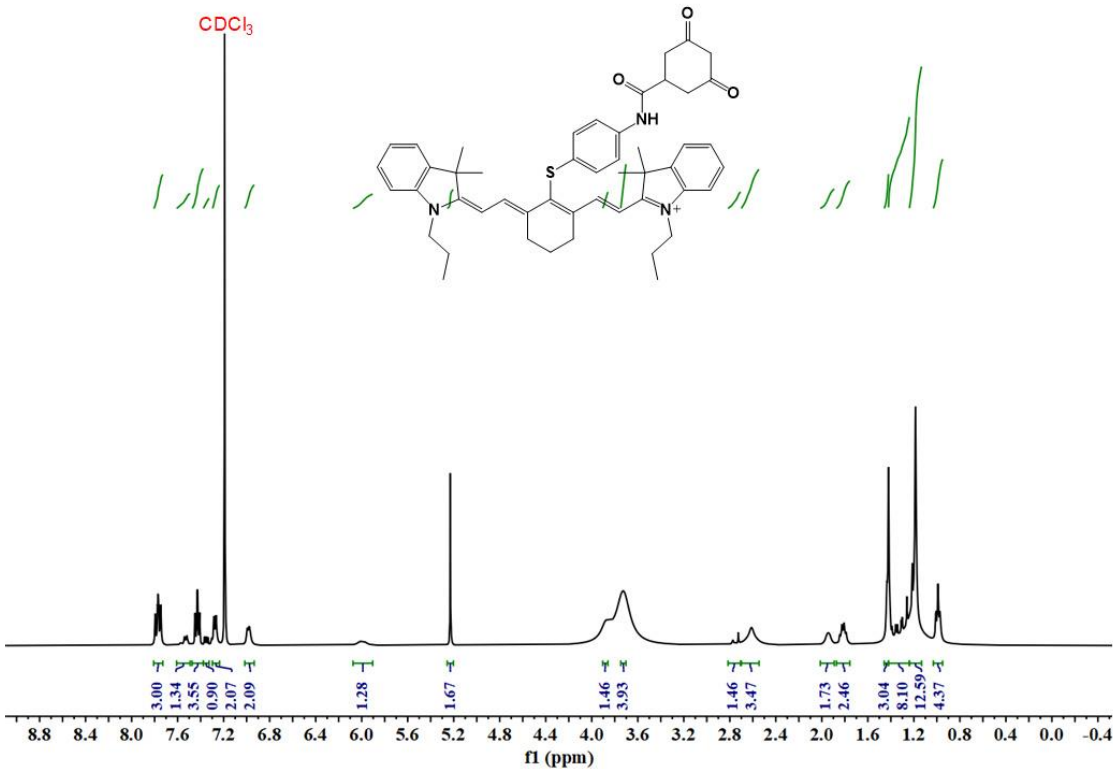

Figure S7. ^{1}H NMR spectrum of DIR. Chloroform-d was used as solvent.

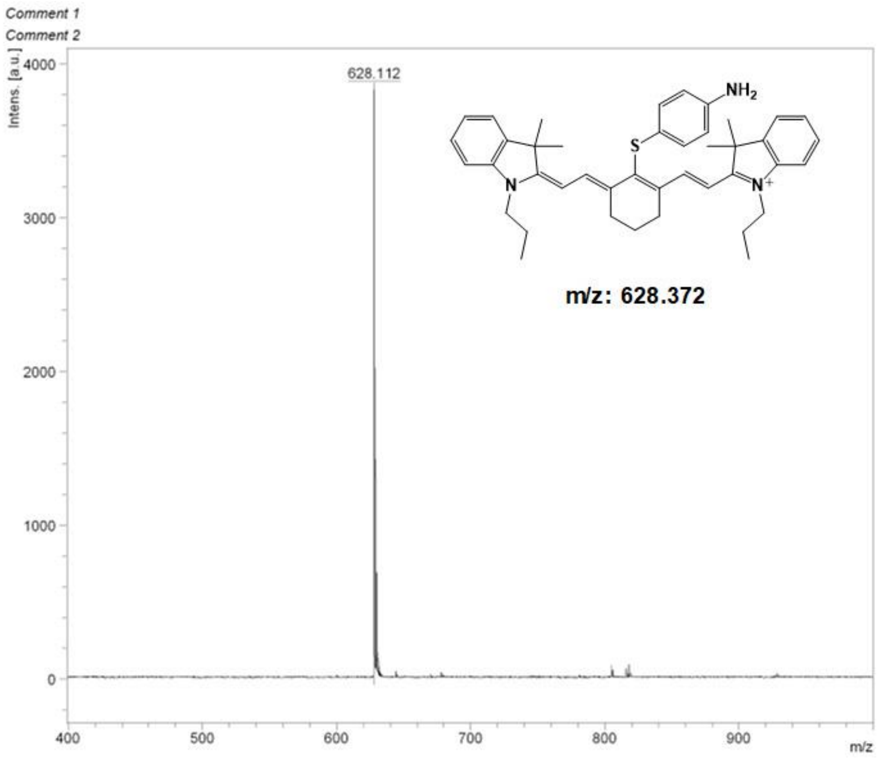

Figure S8. MALDI-TOF-MS spectrum of IR780-NH_2.

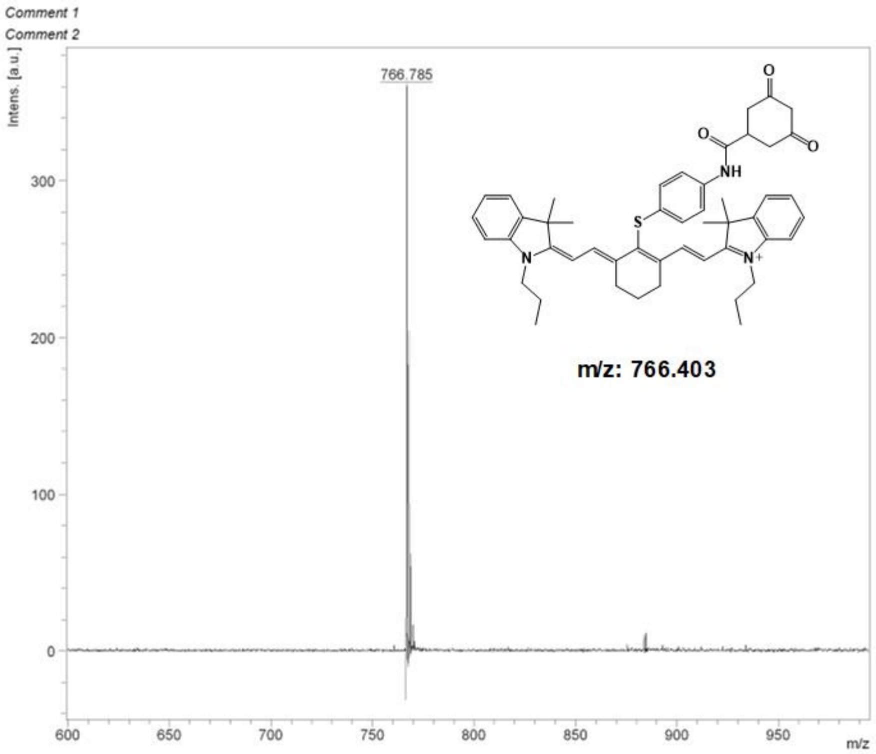

Figure S9. MALDI-TOF-MS spectrum of DIR.

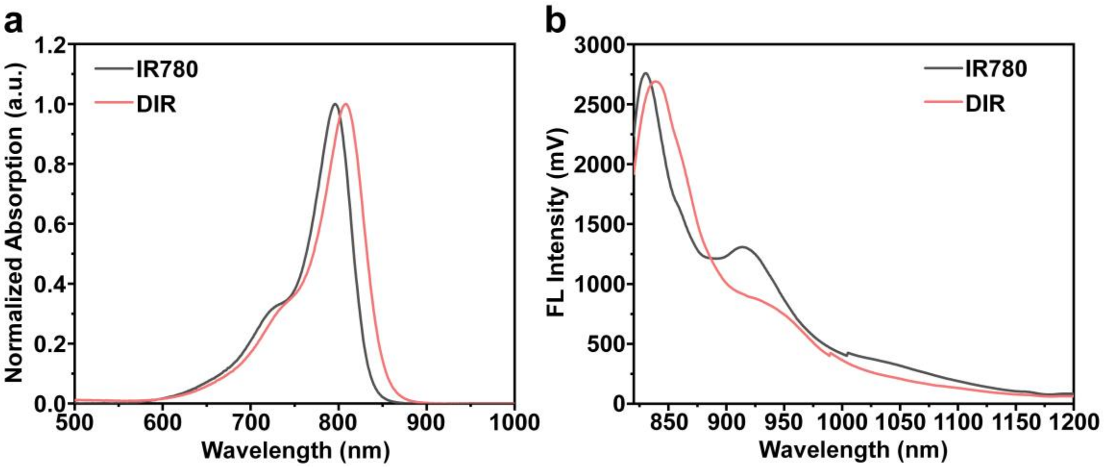

Figure S10. (a) Normalized absorption and (b) fluorescence spectra of IR780 and DIR in DMSO.

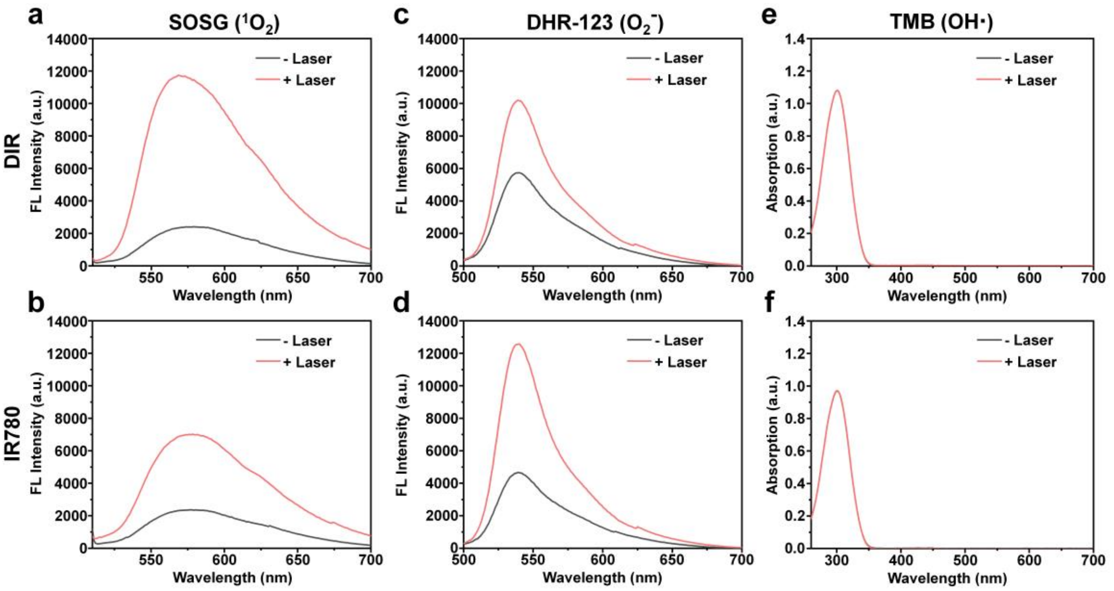

Figure S11. Emission spectra or absorption spectra change of (a, b) SOSG (5 μM), (c, d) DHR-123 (5 μM), and (e, f) TMB (5 μM) in the presence of DIR (20 μM) or IR780 (20 μM) in DMSO under 808 nm laser irradiation (0.1 W cm^{-2}, 1 min).

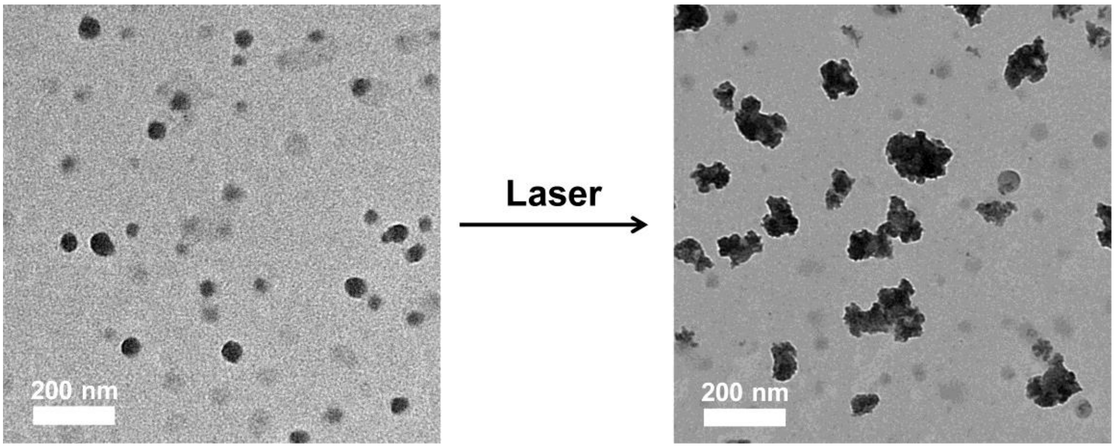

Figure S12. Representative TEM image of DIR NPs with or without 808 nm laser irradiation (0.3 W cm^{-2}, 1 min) in PBS (pH = 7.4). The scale bars represent 200 nm.

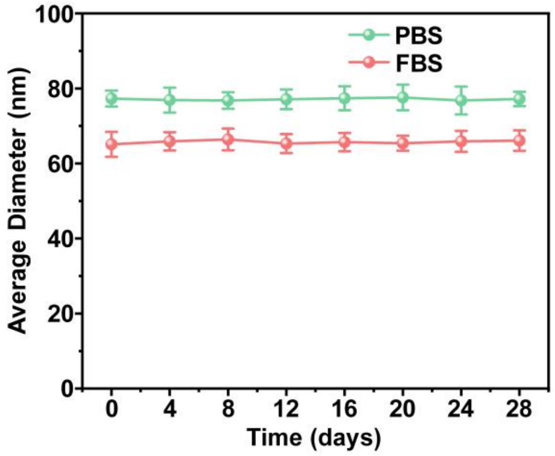

Figure S13. Hydrodynamic size changes of DIR NPs in PBS or 5% FBS with the storage time.

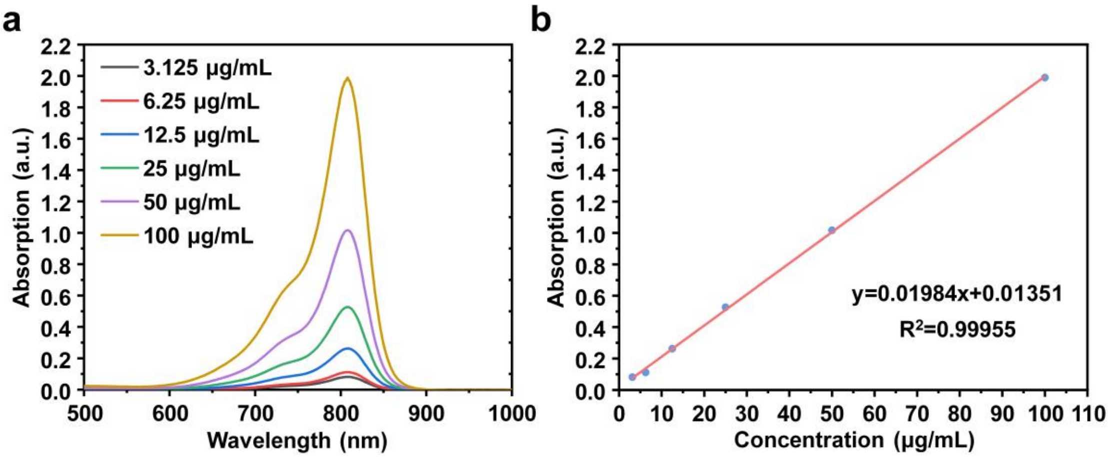

Figure S14. (a) Absorption spectra of DIR NPs with different concentrations. (b) The linear function of absorbance intensity versus concentration at 808 nm.

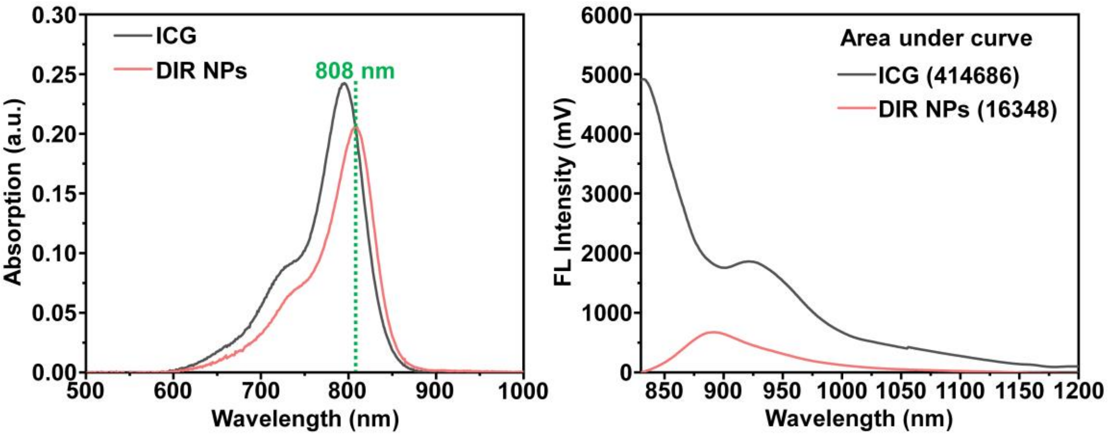

Figure S15. Fluorescence spectra of ICG in DMSO and DIR NPs in water with the

same absorption at 808 nm.

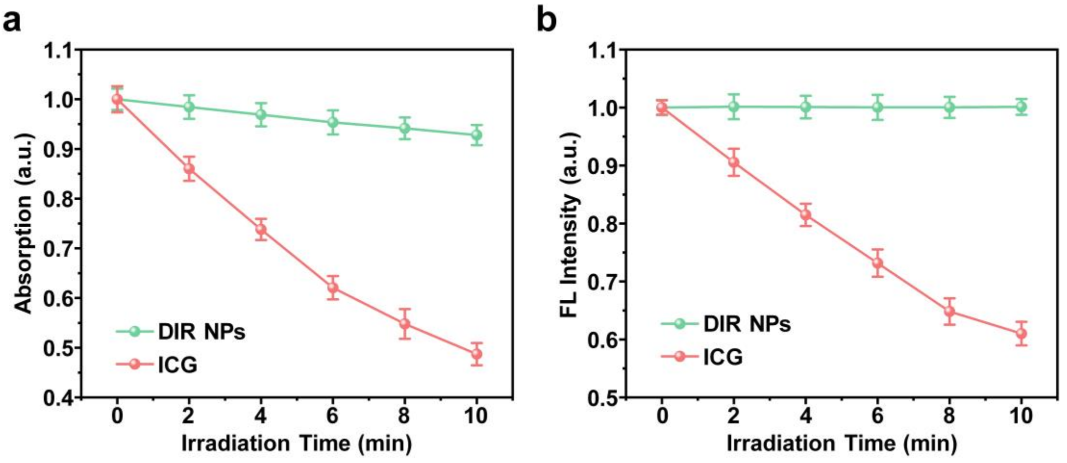

Figure S16. Normalized absorption (a) and fluorescence (b) intensity changes of DIR NPs (20 μg mL^{-1}) and ICG at different laser irradiation time (808 nm, 1 W cm^{-2}).

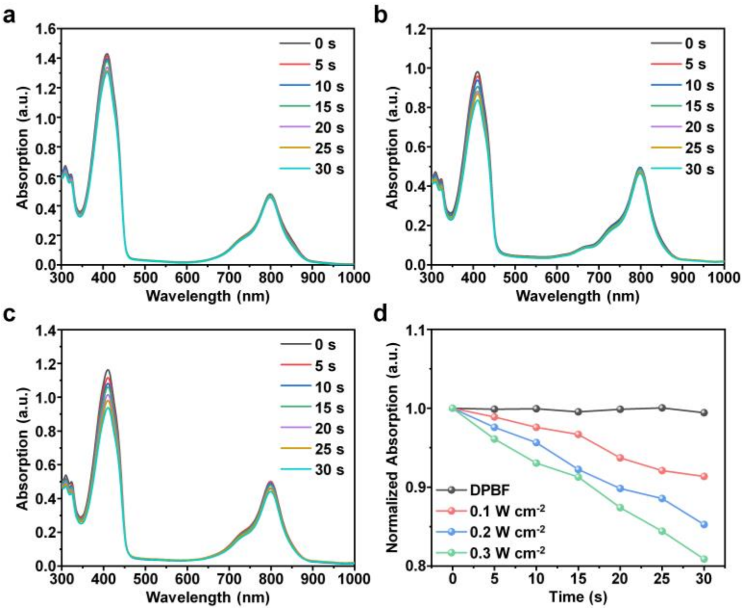

Figure S17. Absorption changes of DPBF incubated DIR NPs (20 μg mL^{-1}) with time under different power of 808 nm laser irradiation (a: 0.1 W cm^{-2}, b: 0.2 W cm^{-2}, c: 0.3 W cm^{-2}). (d) Normalized absorption changes of DPBF incubated DIR NPs with time under 808 nm laser irradiation.

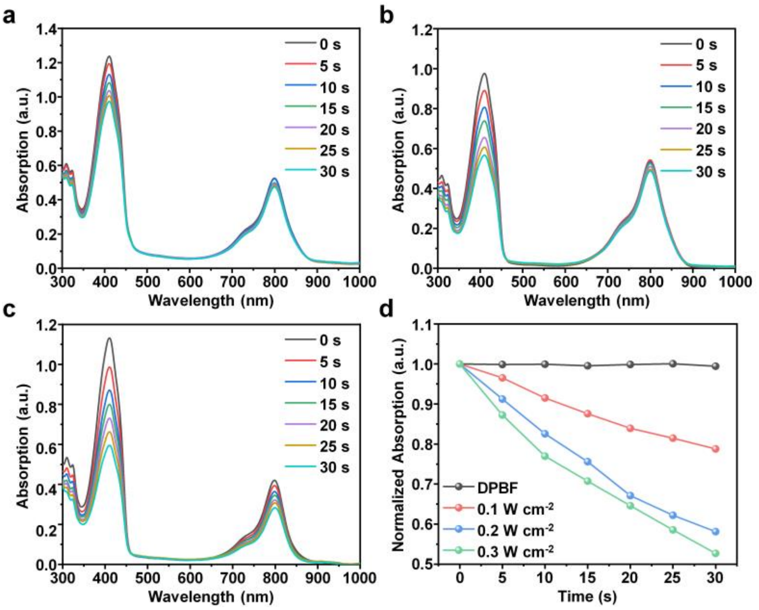

Figure S18. Absorption changes of DPBF incubated DIR NPs (20 μg mL^{-1}) under treatment of BSA-SOH with time under different power of 808 nm laser irradiation (a: 0.1 W cm^{-2}, b: 0.2 W cm^{-2}, c: 0.3 W cm^{-2}). (d) Normalized absorption changes of DPBF incubated DIR NPs with time under 808 nm laser irradiation.

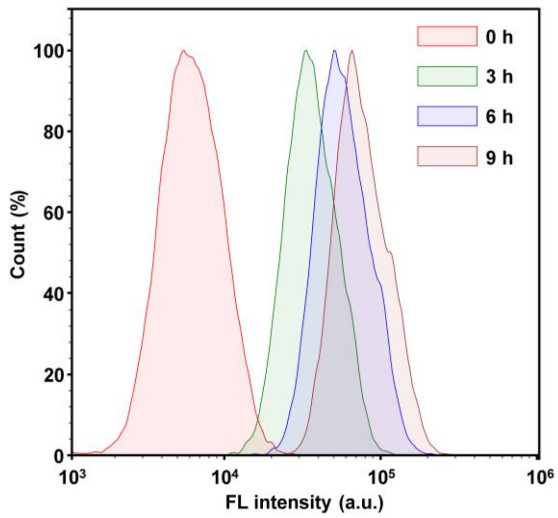

Figure S19. Flow cytometry analysis of 4T1 cells incubated with FITC-loaded DIR NPs for 0 (control), 3, 6, and 9 h.

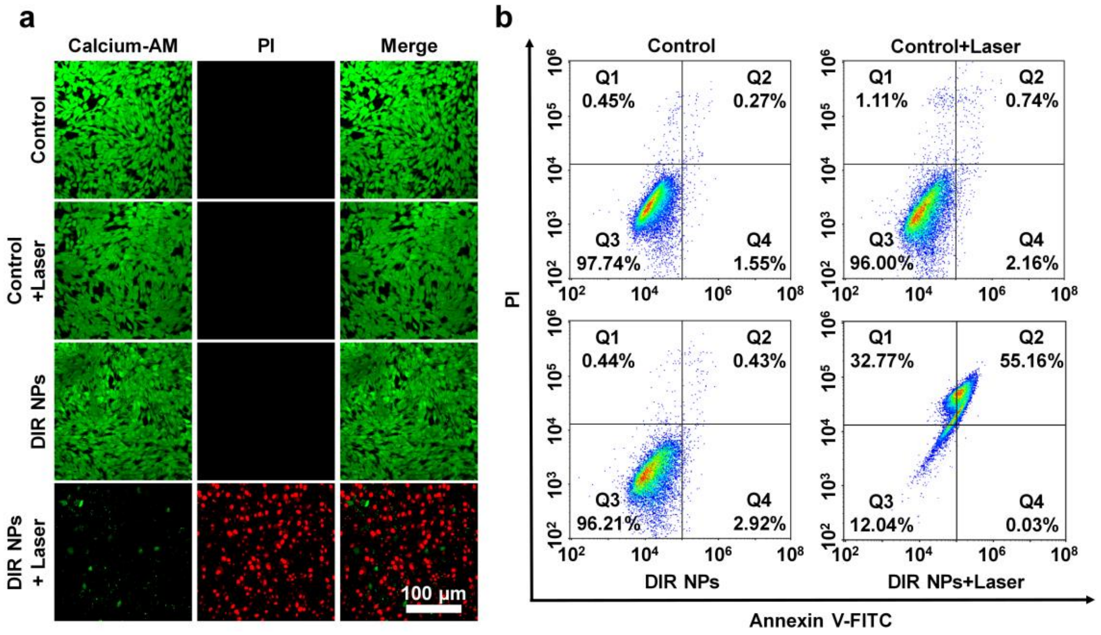

Figure S20. (a) Live/Dead assay of 4T1 cells under different treatments with or without 808 nm laser irradiation (0.3 W cm^{-2}, 5 min). The scale bar represents 100 μm. (b) Representative flow cytometry plots of 4T1 cells under different treatments.

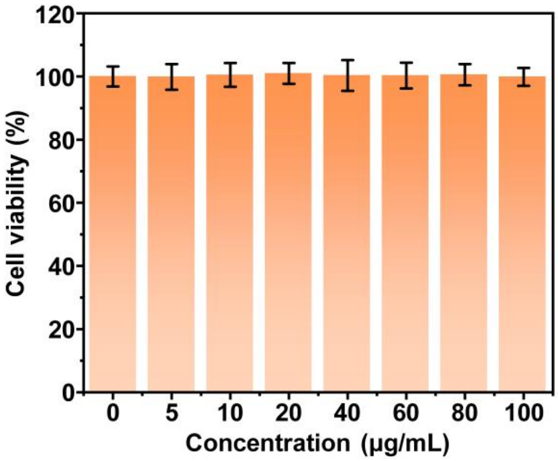

Figure S21. Viability of NIH 3T3 cells treated with different concentrations of DIR NPs without laser irradiation.

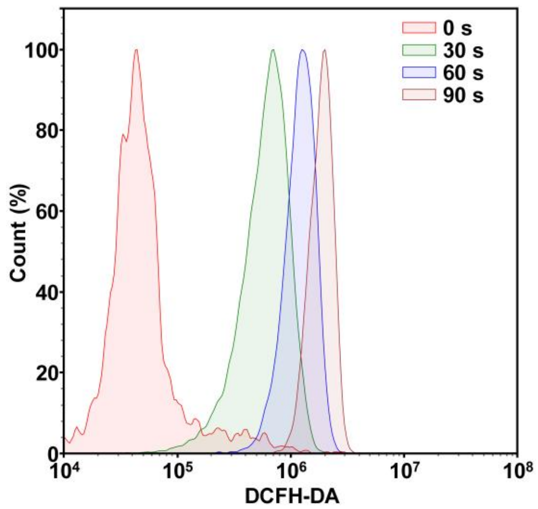

Figure S22. Flow cytometric analysis of intracellular $^1O_2$ production in 4T1 cells treated with DIR NPs (20 μg mL^{-1}) and DCFH-DA (20 μM) after 808 nm laser (0.3 W cm^{-2}) irradiation for 0 (without laser), 30, 60, and 90 s.

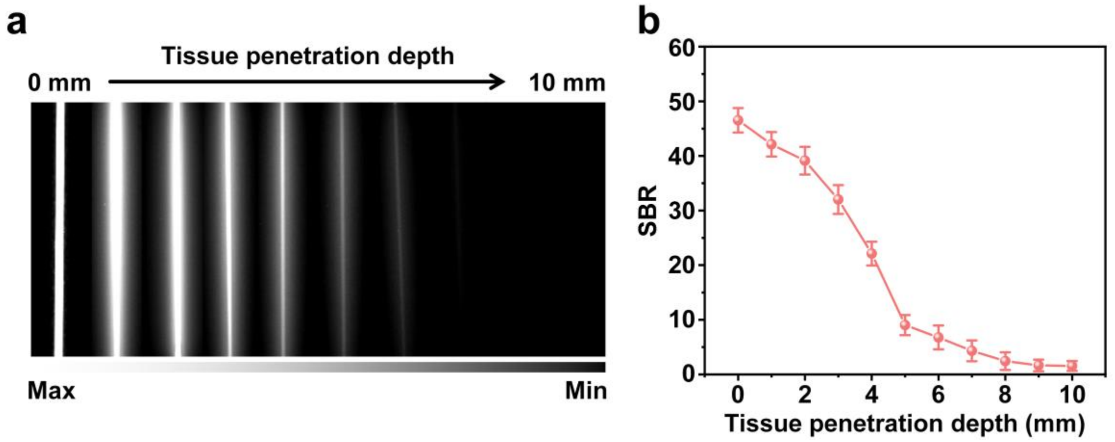

Figure S23. (a) Representative fluorescence images of capillary tubes containing DIR NPs (20 μg mL^{-1}) solutions upon overlaying intralipid with the desired thickness on top of the samples. (b) The signal to background ratios (SBR) for fluorescence imaging of DIR NPs as a function of intralipid depth in (a).

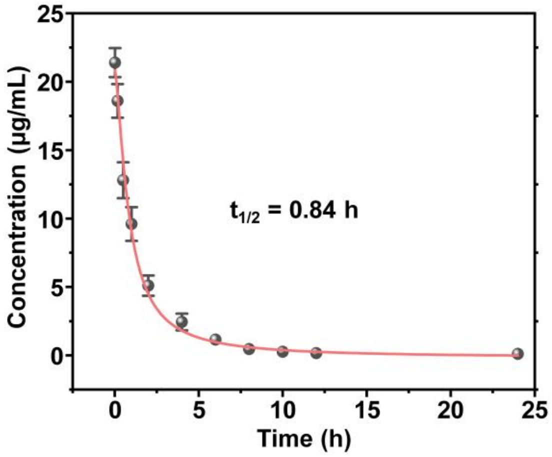

Figure S24. Blood concentration of DIR NPs as a function of postinjection time. The error bars represent standard deviation of three separate measurements (n = 3).

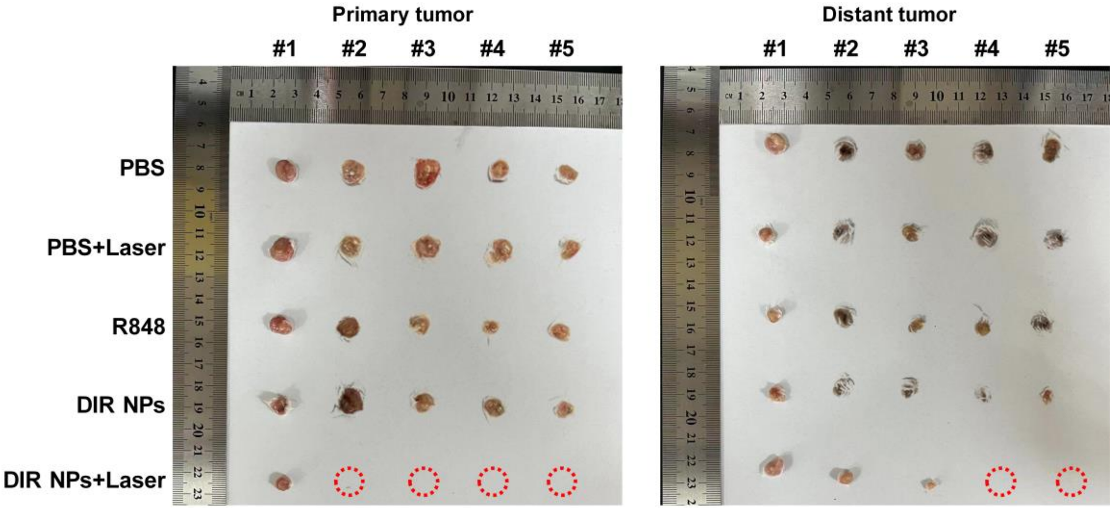

Figure S25. Representative photographs of primary tumors and distant tumors after different treatments for 21 days.

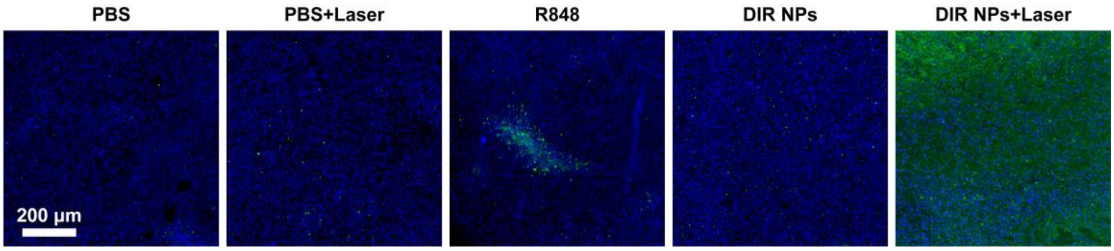

Figure S26. TUNEL staining of primary tumors after different treatments for 21 days. The scale bars represent 200 μm.

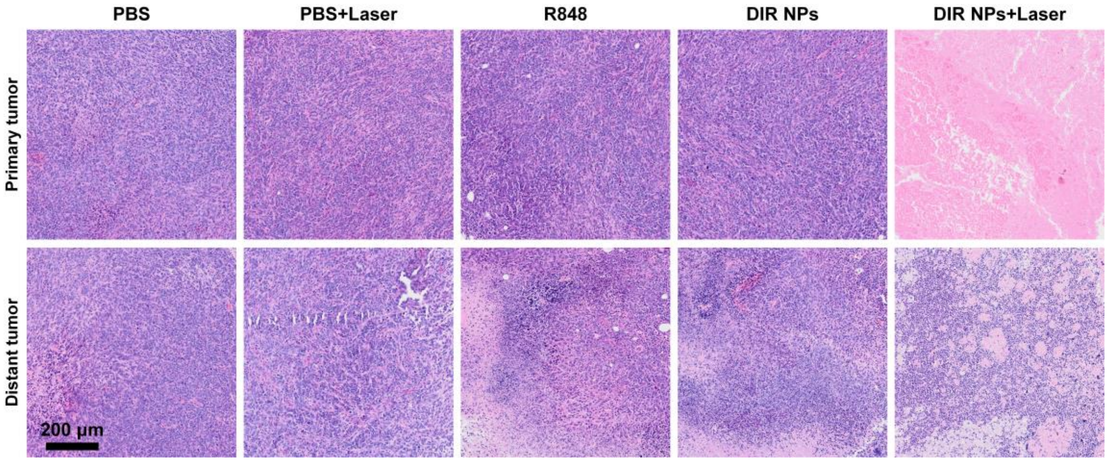

Figure S27. H&E staining of primary tumors and distant tumors after different treatments for 21 days. The scale bars represent 200 μm.

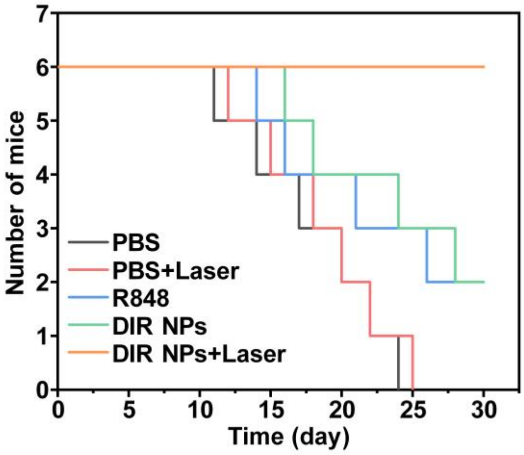

Figure S28. Number of mice survived after different treatments for 30 days.

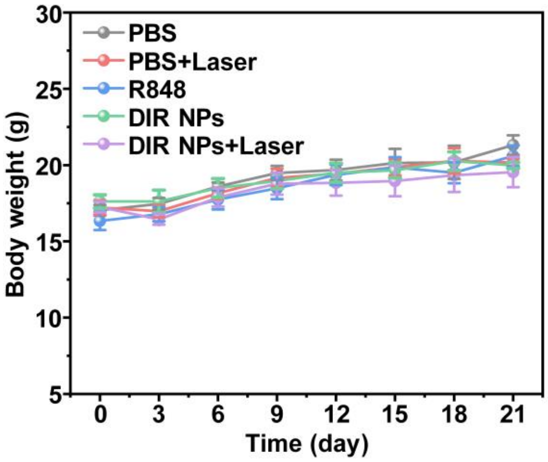

Figure S29. Body weight curves of 4T1 tumor-bearing mice following various treatments.

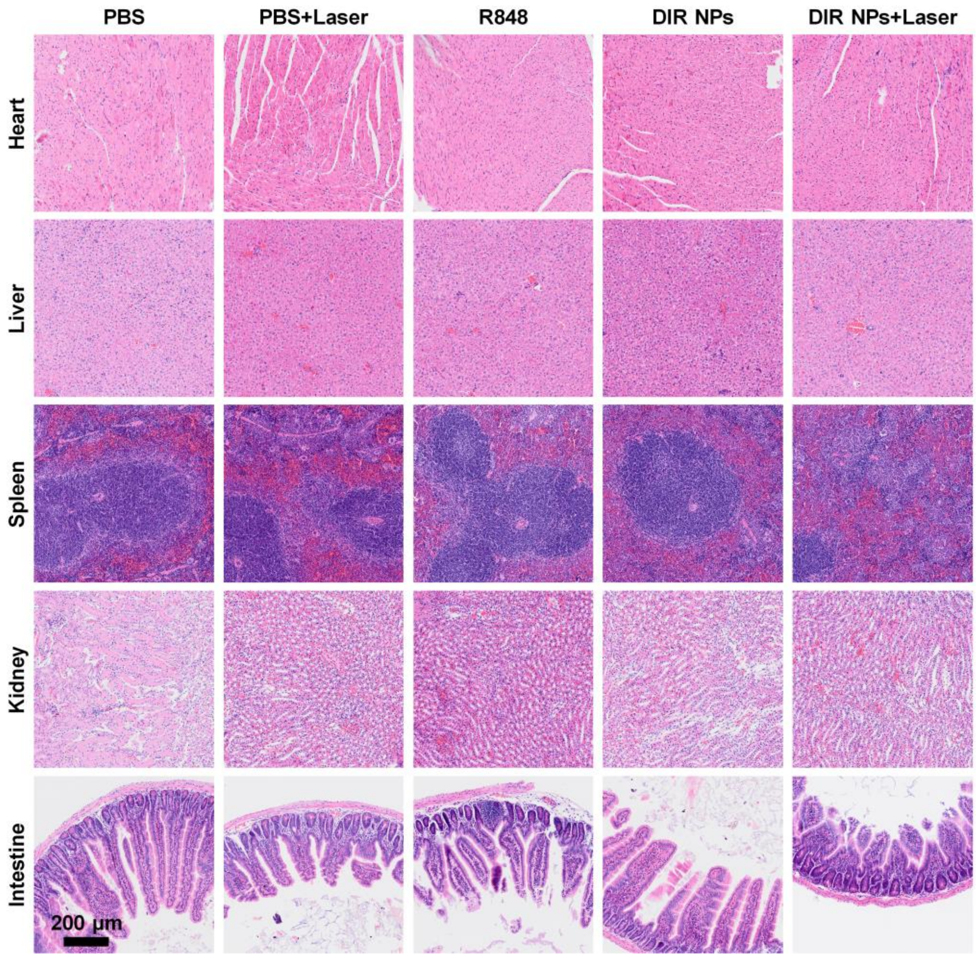

Figure S30. H&E staining of hearts, livers, kidneys, and spleens obtained from 4T1 tumor-bearing mice at day 21 after different treatments. The scale bar represents 200 μm.

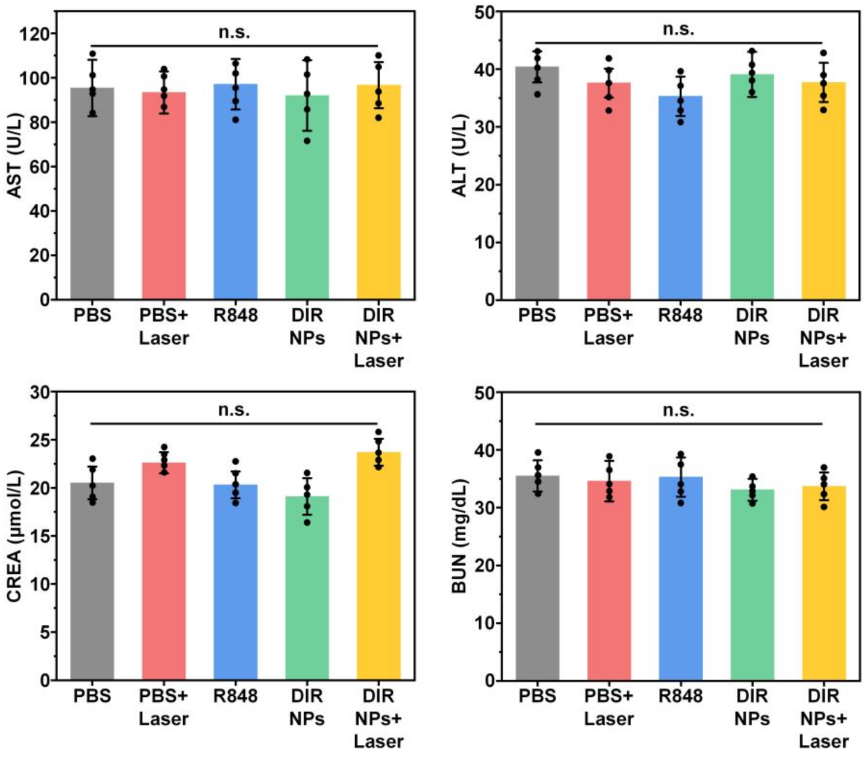

Figure S31. Biochemical analysis of blood collected from mice after various treatments. The error bars represent standard deviations of five different measurements (n = 5). n.s.: not significant.

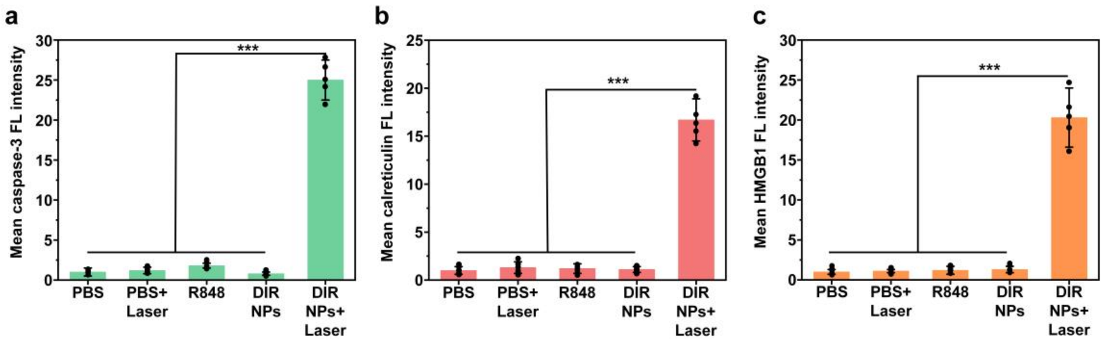

Figure S32. Mean fluorescence intensity of caspase-3 (a), calreticulin (b) and HMGB1 (c) in images from Figure 6 (g), (h) and (i), respectively. The error bars represent the standard deviations of five different measurements (n = 5). ***$p < 0.001$.

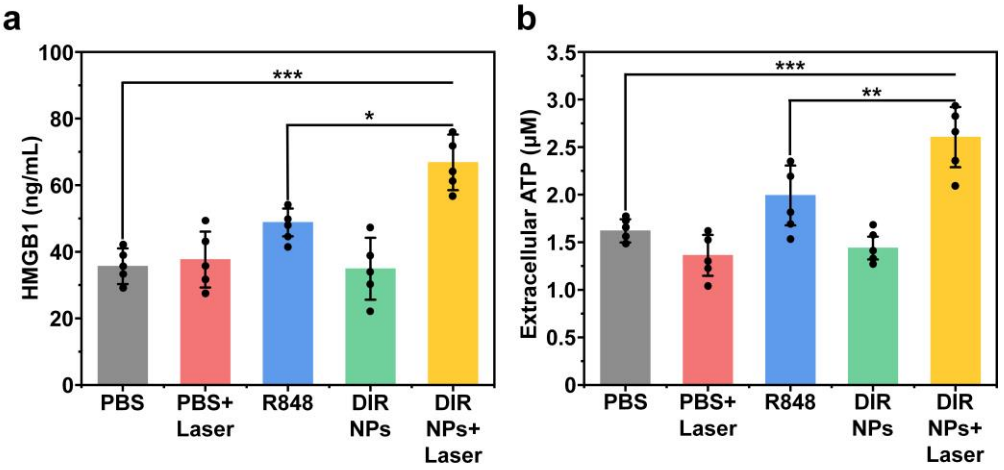

Figure S33. The extracellular levels of HMGB1 (a) and ATP (b) in tumor tissues following laser irradiation detected by ELISA. The error bars represent the standard deviations of five different measurements (n = 5). **p* < 0.05, ***p* < 0.01, ****p* < 0.001.

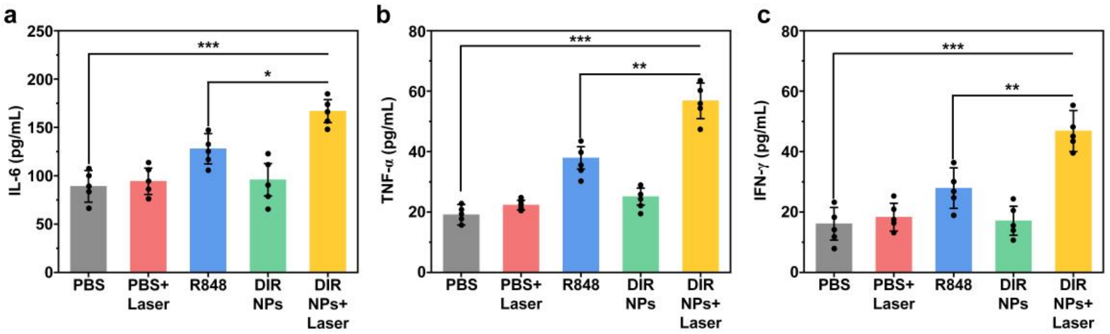

Figure S34. Cytokine levels of IL-6 (a), TNF-α (b) and IFN-γ (c) in serum from different groups after 3 days of treatment. [R848] = 5 mg kg^{-1}, [DIR] = 2 mg kg^{-1}, 808 nm laser irradiation, 0.3 W cm^{-2}, 10 min. The error bars represent standard deviations of five separate measurements (n = 5). **p* < 0.05, ***p* < 0.01, ****p* < 0.001.

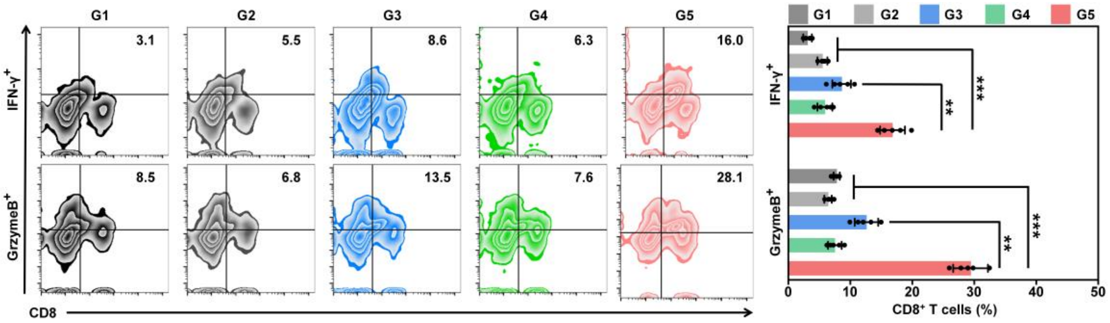

Figure S35. Representative flow cytometric plots and quantitative analysis of IFN γ^{+} T cells and GrzymeB^{+} T cells among CD8^{+} T cell subsets in primary tumor at day 10 after

different treatments. G1, PBS; G2, PBS+Laser; G3, R848; G4, DIR NPs; G5, DIR NPs+Laser.The error bars represent standard deviations of five separate measurements (n = 5). **$p < 0.01$, ***$p < 0.001$.

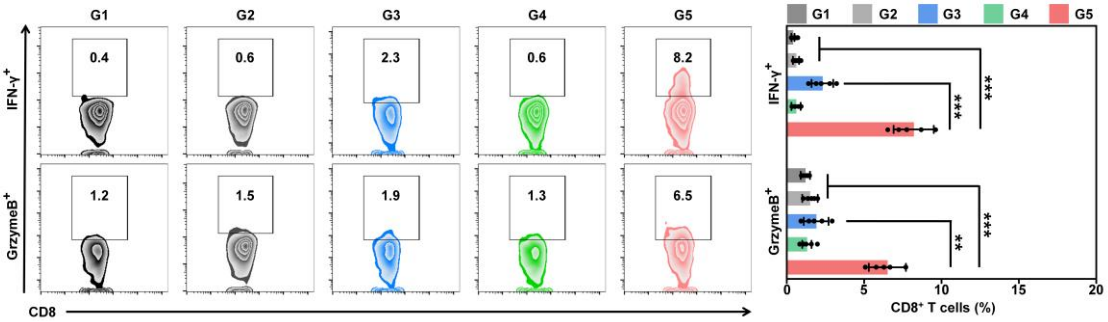

Figure S36. Representative flow cytometric plots and quantitative analysis of IFN γ^+ T cells and GrzymeB$^+$ T cells among CD8$^+$ T cell subsets in draining lymph nodes at day 10 after different treatments. G1, PBS; G2, PBS+Laser; G3, R848; G4, DIR NPs; G5, DIR NPs+Laser. The error bars represent standard deviations of five separate measurements (n = 5). **$p < 0.01$, ***$p < 0.001$.

2. Experimental section

2.1 Materials and Characterizations.

Materials. All chemicals in the experiments were received from Sigma-Aldrich and used without further purification unless otherwise mentioned. RPMI 1640 medium (Catalog: KGM31800), Dulbecco's Modified Eagle Medium (DMEM, Catalog: KGM12800), fetal bovine serum (FBS), 4,6-Diamidino-2-phenylindole (DAPI, Catalog: KGA215), MTT cell proliferation and Cytotoxicity Detection Kit (Catalog: KGA311), 2',7'-Dichlorodihydrofluorescein diacetate (DCHF-DA, Catalog: KGAF018), Annexin V-FITC/PI Apoptosis Detection Kit (Catalog: KGA108) and Live & Dead Viability/Cytotoxicity Assay Kit (Catalog: KGAF001) were purchased from Jiangsu KeyGEN Biotech Corp., Ltd. (Nanjing, China). ATP Assay Kit (Catalog: S0027), Calreticulin Rabbit Monoclonal Antibody (Catalog: AF1666), HMGB1 Rabbit Polyclonal Antibody (Catalog: AF0180), Caspase-3 Rabbit Polyclonal Antibody (Catalog: AF0081), Alexa Fluor 488-labeled Goat Anti-Rabbit IgG (H+L) (Catalog: A0423), Mouse ELISA kits for TNF-α (Catalog: PT512), IFN-γ (Catalog: PI507), and

IL-6 (Catalog: PI326) were purchased from Beyotime Biotechnology Company (Shanghai, China). Mouse HMGB1 ELASI Kit was purchased from Elabscience (Catalog: E-EL-M0676). Anti-mouse CD16/32 (clone: 93), FITC anti-mouse CD11c (clone: N418), APC anti-mouse CD80 (clone: 16-10A1), PE anti-mouse CD86 (clone: GL-1), BV510 anti-mouse CD45 (clone: 30-F11), APC anti-mouse CD3 (clone: 17A2), PE-Cy7 anti-mouse CD4 (clone: RM4-5), BV650 anti-mouse CD8 (clone: 53-6.7), FITC anti-mouse CD44 (clone: IM7), and PE anti-mouse CD62L (clone: MEL-14) were purchased from Biolegend (San Diego, USA). Recombinant murine granulocyte-macrophage colony-stimulating factor (GM-CSF, Catalog: 315-03) and interleukin-4 (IL-4, Catalog: 214-14) were purchased from PeproTech (Rocky Hill, USA).

Characterizations. Proton nuclear magnetic resonance (^{1}H NMR) was conducted on a Bruker Ultra Shield Plus 400 MHz Spectrometer. $CDCl_3$ and MeOH-d_4 were used as the solvents. The matrix-assisted laser desorption/ionization time-of-flight (MALDI-TOF) mass spectra were measured by a Bruker Autoflex TOF/TOF spectrometer. Transmission electron microscopy (TEM) measurements were carried out using a JEOL JEM-2100 transmission electron microscope operating at an acceleration voltage of 100.0 kV. Dynamic light scattering (DLS) and zeta potential results were measured on a NanoBrook ZetaPALS Potential Analyzer. The UV-visible absorption spectra were obtained on a Shimadzu UV-3600 Plus spectrophotometer. Fluorescence spectra were recorded with a HORIBA Fluoromax-4 fluorescence spectrometer. Confocal fluorescence images were captured on a LSM880 confocal laser scanning microscope (Carl Zeiss, Germany). Flow cytometry analysis was conducted on a Flow Sight Imaging Flow Cytometer (Merck Millipore, Darmstadt, Germany). *In vivo* NIR-II fluorescence imaging was carried out on a NIR-II imaging system (Wuhan Grand-imaging Technology Co., Ltd). All the mice experiments were carried out in accordance with the guidelines of the Laboratory Animal Center of Jiangsu KeyGEN Biotech Corp., Ltd and approved by the Animal Ethics Committee of Simcere BioTech Corp., Ltd.

2.2 Synthesis of Compound 1. Acetone (5.9 g, 100 mmol) and thioglycolic acid (19.6 g, 210 mmol) were dissolved into 50 mL DCM, then 10 mL of trifluoroacetic acid (TFA) was added to the solution. The resulting solution was stirred at room temperature for

24 h. DCM was then removed by distillation under reduced pressure. A large amount of white solid was precipitated after adding 100 mL of petroleum ether, which was filtered off with suction and washed with petroleum ether and water three times, and dried in a vacuum drying oven to give compound 1 (20.6 g, yield 91.6%). ^{1}H NMR (400 MHz, MeOH-d4, δ): 3.32 (s, 4H), 1.51 (s, 6H).

2.3 Synthesis of Compound 2. Under the ice bath, 15.4 mL $LiAlH_4$ in tetrahydrofuran (THF, 2.4 mol/L) was added dropwise into 20 mL dry THF solution containing 5 g compound 1 and stirred for 1 h, then the reaction was carried out at room temperature for another 1 h. Then 15% NaOH aqueous solution was slowly added into the reaction mixture until no gas was generated. After that, the mixture was filtered, and the filtrate was distilled under reduced pressure. The crude product was further purified by silica gel column chromatograph using dichloromethane (DCM)/methanol (MeOH) (20:1, v/v) as the eluent, and hydroxyl-terminal ROS-cleavable TK linker (TK-OH, compound 2) was obtained as viscous liquid (2.4 g, the yield is 54.8%). ^{1}H NMR (400 MHz, MeOH-d_4, δ): 3.70 (t, J = 7.0 Hz, 4H), 2.80 (t, J = 7.0 Hz, 4H), 1.61 (s, 6H).

2.4 Synthesis of Compound 3. Compound 2 (0.10 g, 0.89 mmol), 5-hexynoic acid (0.70 g, 3.57 mmol), DMAP (0.05 g, 0.40 mmol) were added to 5 mL of anhydrous THF, then 0.08 g of DIC was added and the resulting solution was stirred at room temperature for 24 h. After the reaction, the THF was removed by distillation under reduced pressure, 10 mL of DCM was added to dissolve the residue. The solution was washed with saturated brine for three times, dried with anhydrous Na_2SO_4. The crude product was purified by silica gel column with DCM/MeOH (40:1, v/v) as the eluent (155.6 mg, the yield is 60.1%). ^{1}H NMR (400 MHz, MeOH-d4, δ): 4.33 (t, J = 6.8 Hz, 1H), 4.14 (t, J = 6.9 Hz, 2H), 3.59 (t, J = 6.9 Hz, 2H), 2.79 (t, J = 6.9 Hz, 2H), 2.68 (t, J = 6.9 Hz, 2H), 2.37 (t, J = 7.4 Hz, 2H), 2.13 (t, J = 2.4 Hz, 2H), 1.71 (dd, J = 5.7, 2.3 Hz, 2H), 1.50 (s, 6H).

2.5 Synthesis of Compound 4. Compound 3 (0.10 g, 0.34 mmol) was dissolved in 10 mL dry DCM and cooled to 0 °C, then 104 mg triethylamine (TEA) was added and stirred for 5 min. Then, 4-nitrobenzoyl chloride (0.076 g, 0.38 mmol) was slowly added to the mixture and stirred at 0 °C for 1 h. After the reaction, the solution was washed

with saturated $NaHCO_3$ (3×50 mL) and dried with anhydrous Na_2SO_4. The crude product was obtained by silica gel column with DCM/PE (1:1, v/v) as the eluent (127.9 mg, the yield is 81.5%). ^{1}H NMR (400 MHz, $CDCl_3$, δ): 8.26 – 8.18 (m, 2H), 7.36 – 7.30 (m, 2H), 4.38 (t, J = 7.0 Hz, 2H), 4.19 (t, J = 6.9 Hz, 2H), 2.93 (t, J = 7.0 Hz, 2H), 2.82 (t, J = 6.9 Hz, 2H), 2.40 (t, J = 7.4 Hz, 2H), 2.20 (td, J = 7.0, 2.7 Hz, 2H), 1.78 (p, J = 7.2 Hz, 2H), 1.57 (s, 6H).

2.6 Synthesis of Compound 5. Resiquimod (R848, 0.076 g, 0.24 mmol) was dissolved in 5 mL dry DMF, then diisopropylethylamine (DIPEA, 0.063 g, 0.48 mmol) was added dropwise, and the solution was stirred at room temperature for 5 min. To this mixture was added the compound 4 (0.10 g,0.22 mmol) in one portion, and the reaction mixture immediately turned bright yellow. The vial was sealed and heated at 60 °C and stirred for 16 h. The reaction mixture was then cooled, and EtOAc (40 mL) was added. The organic layer was washed with H_2O (3×100 mL) and brine (1×100 mL), dried over anhydrous Na_2SO_4, filtered, and the EtOAc was removed in vacuo. The crude product was purified by silica gel column with DCM/MeOH (30:1, v/v) as the eluent (101.7 mg, the yield is 73.5%). ^{1}H NMR (400 MHz, $CDCl_3$, δ): 8.08 (dd, J = 8.4, 1.4 Hz, 1H), 7.54 (ddd, J = 8.4, 7.0, 1.3 Hz, 1H), 7.41 (ddd, J = 8.4, 7.0, 1.4 Hz, 1H), 7.01 (dd, J = 8.5, 2.5 Hz, 1H), 4.85 (s, 2H), 4.73 (s, 2H), 4.37 (t, J = 7.0 Hz, 2H), 4.18 (t, J = 6.9 Hz, 2H), 3.60 (q, J = 7.0 Hz, 2H), 2.92 (t, J = 7.0 Hz, 2H), 2.83 (t, J = 6.8 Hz, 2H), 2.39 (t, J = 7.4 Hz, 2H), 2.19 (td, J = 7.0, 2.7 Hz, 2H), 1.81 – 1.74 (m, 2H), 1.58 (s, 6H), 1.30 (s, 6H), 1.26 (s, 3H).

2.7 Synthesis of $mPEG_{2k}$-TK-R848. Compound 5 (0.10 g), $mPEG_{2k}$-N_3 (0.42 g) and PMDETA (30 mg) were placed in a Schlenk tube, and 3 mL of anhydrous THF was added to dissolve the solids. The obtained solution was degassed via three freeze-pump-thaw cycles after addition of 20 mg of CuBr. The reaction was carried out at room temperature for 24 h. After that, 30 mL of deionized water was added to the tube, and the resulting solution was dialyzed against pure water for 3 days. The product $mPEG_{2k}$-TK-R848 was obtained after lyophilization (128.7 mg).

2.8 Synthesis of IR780-NH_2. IR780 (0.10 g, 0.15 mmol) and 4-aminothiophenol (28.1 mg, 0.24 mmol) were dissolved in 5 mL anhydrous DCM, and stirred at room

temperature for 24 h. After that, the mixture were extracted three times with saturated brine, dried with anhydrous Na_2SO_4, and the crude product was purified by a silica gel column with DCM/MeOH (30:1, v/v) as the eluent (88.9 mg, yield 94.3%).^{1}H NMR (400 MHz, $CDCl_3$, δ): 8.66 (d, J = 14.2 Hz, 2H), 7.32 – 7.22 (m, 4H), 7.16 – 7.11 (m, 2H), 7.03 (s, 2H), 7.01 – 6.94 (m, 2H), 6.77 (d, J = 8.3 Hz, 2H), 6.12 (d, J = 14.1 Hz, 2H), 5.23 (s, 1H), 4.03 (t, J = 7.3 Hz, 3H), 3.93 (t, J = 7.4 Hz, 1H), 2.66 (t, J = 6.1 Hz, 3H), 2.60 (t, J = 6.0 Hz, 1H), 1.94 (d, J = 7.1 Hz, 2H), 1.83 (q, J = 7.2 Hz, 4H), 1.48 (s, 9H), 1.43 (s, 3H), 1.19 (s, 4H), 0.99 (td, J = 7.4, 4.3 Hz, 6H). MS (MALDI-TOF, m/z) Calcd for $C_{42}H_{50}N_3S^+$, 628.372; Found: 628.112.

2.9 Synthesis of DIR. IR780-NH_2 (0.10 g, 0.16 mmol), DHCA (0.027 g, 0.17 mmol), HATU (0.20 g, 0.53 mmol), HOBT (0.07 g, 0.53 mmol), DIPEA (0.07 g, 1.06 mmol) were dissolved into 5 mL anhydrous DMF. The reaction was carried out at room temperature for 24 h. DMF was removed by distillation under reduced pressure, and 10 mL of DCM was added to dissolve the residue. The resulting solution was extracted with saturated brine for three times and dried with anhydrous Na_2SO_4. The crude product was purified by silica gel column with DCM/MeOH (50:1, v/v) as the eluent (80.4 mg, yield 79.3%).^{1}H NMR (400 MHz, $CDCl_3$, δ): 7.81 – 7.73 (m, 3H), 7.61 – 7.49 (m, 1H), 7.43 (td, J = 7.4, 1.3 Hz, 4H), 7.35 (dd, J = 8.4, 4.6 Hz, 1H), 7.28 (d, J = 7.5 Hz, 2H), 6.99 (d, J = 7.7 Hz, 2H), 6.00 (s, 1H), 5.23 (s, 2H), 3.88 (s, 1H), 3.73 (s, 4H), 2.75 (d, J = 19.2 Hz, 1H), 2.61 (s, 3H), 1.94 (s, 2H), 1.82 (q, J = 7.4 Hz, 2H), 1.44 (s, 3H), 1.42 – 1.24 (m, 8H), 1.20 (d, J = 11.2 Hz, 13H), 0.99 (t, J = 7.3 Hz, 4H). MS (MALDI-TOF, m/z) Calcd for $C_{49}H_{56}N_3O_3S^+$, 766.403; Found: 766.785.

2.10 Preparation and Characterization of DIR NPs. DIR NPs were prepared by a nanoprecipitation method. DIR (1 mg) and $mPEG_{2k}$-TK-R848 (20 mg) were dissolved into 1 mL of THF. The obtained solution was rapidly injected into a mixture of 10 mL of water and 1 mL of THF under vigorous sonication for 3 min. The resulting nanoparticle solutions were placed under a gentle nitrogen flow to remove THF. The obtained THF-free solution was filtered through a 0.22 μm PVDF syringe driven filter. The purified nanoparticle solutions were concentrated via ultracentrifugation and stored at 4 °C.

2.11 Measure of fluorescence quantum yields.[1] Indocyanine green was used as a standard with a known fluorescence quantum yield (Φ) value of 13% in DMSO. Fluorescence quantum yields were calculated using the following equation: $\frac{\Phi_s}{\Phi_f} = \left(\frac{A_s}{A_f}\right) \times \left(\frac{Abs_s}{Abs_f}\right) \times (\frac{\eta_s^2}{\eta_f^2})$ where Φ_s and Φ_f are the fluorescence quantum yields of the standard and the samples, respectively; A_s and A_f are the emission areas of the standard and the samples, respectively; Abs_s and Abs_f are the absorbance of the standard and the samples at the Wavelength of excitation; η_s and η_f are the refractive indices of the standard and the samples, respectively.

2.12 In vitro tissue-penetration study of DIR NPs. Glass capillary tubes were filled with DIR NPs (20 μg mL^{-1}). Fluorescence images of capillary tubes containing DIR NPs covered with varied thicknesses of 1 % Intralipid were captured. This is because 1% Intralipid had similar scattering characteristics to tissue samples.[2] The NIR-II fluorescence images were acquired with excitation at 808 nm and emission at 980 nm long pass filter and an acquisition time of 50 ms.

2.13 Measurement of singlet oxygen generation. Singlet oxygen generation of DIR NPs under 808 nm laser irradiation were measured based on monitoring the absorption loss of 1,3-diphenylisobenzofuran (DPBF). DPBF were added into DIR NPs (20 μg mL^{-1}) solution with or without BSA-SOH (10 μg mL^{-1}) to a final concentration of 5 μg mL^{-1}. The obtained solution was irradiated under 808 nm laser with different power (0.1 W cm^{-2}, 0.2 W cm^{-2}, 0.3 W cm^{-2}), and the absorbance at 414 nm was monitored.

2.14 *In vitro* laser-activated R848 release. To determine the laser-activated R848 release, DIR NPs (30 μg mL^{-1}, pH = 7.4) were divided into two groups and placed in a 37 °C incubator. One group was irradiated with 808 nm laser (0.3 W cm^{-2}) at 0, 20, 40, 60, 80, 100 min for 10 min, while the other group was treated without laser. 50 μL of DIR NPs solutions were withdrawn every 10 min. The released R848 was determined by HPLC.

2.15 Cells and animals. 4T1 murine mammary cancer cells (4T1 cells) and NIH 3T3 cells were purchased from Jiangsu KeyGEN Biotech Corp., Ltd. These cells were maintained in a RPMI 1640 medium supplemented with 10 % FBS and 1%

penicillin/streptomycin and incubated at 37 °C, 5% CO_2 in humidified incubators. Five-week-old female BALB/c mice were purchased from Jiangsu KeyGEN Biotech Corp., Ltd. All the mice experiments were carried out in accordance with the guidelines of the Laboratory Animal Center of Jiangsu KeyGEN Biotech Corp., Ltd and approved by the Animal Ethics Committee of Simcere BioTech Corp., Ltd.

2.16 Isolation and culture of bone marrow-derived dendritic cells. Bone marrow-derived dendritic cells (BMDCs) were isolated from the hind limbs of female BALB/c mice. The BMDCs culture medium was RPMI 1640 medium containing 10% heat-inactivated FBS, 1% penicillin/streptomycin, GM-CSF (20 ng mL^{-1}) and IL-4 (5 ng mL^{-1}). The cells were seeded into 24-well plates at a density of 1×10^6 cells mL^{-1} and cultured in a CO_2 incubator (37 °C, 5% CO_2). The culture medium was replaced with the fresh medium supplemented with GM-CSF and IL-4 to remove the unattached cells and cell debris on day 3. Semi-suspended cells and loosely adherent cells were collected for further study between days 7 and 9.

2.17 *In vitro* cellular uptake. Fluorescein isothiocyanate (FITC) was doped into DIR NPs by using similar nanoprecipitation method described in the above to endow them with green fluorescence for confocal imaging. The 4T1 cells were seeded into confocal dishes and cultured in incubator overnight. Then, FITC-loaded DIR NPs were added and cultured with cells for 0, 3, 6 and 9 h. Thereafter, the treated cells were gently washed with fresh PBS, and DAPI was used to stain the cell nuclei. The confocal images were captured under excitation of 405 nm and 488 nm for DAPI and FITC, respectively. For flow cytometry analysis, 4T1 cells were seeded into 6-well cell culture plates (2×10^5 cells per well) and incubated for 0, 3, 6 and 9 h. The cells were then washed with fresh PBS and trypsinized, and collected into 1.5 mL tubes. The fluorescence of cells was detected on a flow cytometry system.

2.18 Cytotoxicity assay (MTT). 4T1 cells and NIH 3T3 cells were seeded into 96-well plates (10000 cells in 100 μL medium per well) and incubated for 24 h. DIR NPs were diluted with fresh medium to different concentrations ([DIR] = 0, 5, 10, 20, 40, 60, 80 and 100 μg mL^{-1}), and then incubated with cells for 24 h and maintained in a humidified 37 °C incubator with 5% CO_2. After incubation for 24 h, 4T1 cells were treated without

or with 808 nm laser irradiation (0.3 W cm^{-2}) for 1 min, for NIH 3T3 cells no laser irradiation was required. The relative cell viabilities were eventually determined by the standard MTT assay as follows. The original medium was replaced by fresh medium with MTT (20 μL, 5 mg mL^{-1}). The cells were incubated in a humidified 37 °C incubator with 5% CO_2 for 4 h. The medium was removed and DMSO (100 μL) was added into each well. The 96-well was fiercely shaken for 5 min at room temperature. The absorbance of assays at 450 nm was measured. Cell viability was calculated by the ratio of the absorbance of the cells incubated with DIR NPs to that of the cells incubated with untreated culture medium. IC_{50} was calculated for the drug concentration from the concentration-dependent cell viability data, in which cell growth was inhibited by 50%.

2.19 Live/Dead assay. 4T1 cells (~5 × 10^4) were seeded into a cell culture dish and allowed to grow for 24 h, and the cells were randomly divided into four groups, which were (1) control, (2) control + laser, (3) DIR NPs, (4) DIR NPs + Laser. For dead/live staining, 4T1 cells were incubated with DIR NPs (20 μg mL^{-1}) for 24 h. 4T1 cells without DIR NPs were used as control. Then, the medium was removed, and washed with fresh PBS buffer three times. Fresh DMEM medium (1 mL) was added to dishes, and 4T1 cells in each dish were treated with laser irradiation for 5 min (808 nm, 0.3 W cm^{-2}). After irradiation, the medium was removed carefully, and 1 μM Calcein AM and 1 μM PI were added into each dish and incubated for 30 min. The medium was then removed, washed with fresh PBS three times, and the fluorescence images of Calcein AM and PI were obtained on a LSM880 confocal laser scanning microscope.

2.20 Apoptosis evaluation by flow cytometry. 4T1 cells (~5 × 10^5) were seeded into 6-well plates and incubated at 37 °C for 24 h, and the cells were randomly divided into four groups, which were (1) control, (2) control + laser, (3) DIR NPs, (4) DIR NPs + Laser. After removal of medium, 4T1 cells were incubated with DIR NPs (20 μg mL^{-1}) for 24 h. 4T1 cells without DIR NPs were used as control. After incubation, the medium was removed and washed with fresh PBS, and fresh culture medium was then added. The cells were then treated with or without 808 nm laser irradiation (0.3 W cm^{-2}) for 5 min. After removal of the medium, the cells were trypsinized, collected, resuspended in 0.2 mL PBS, and stained with Annexin V-FITC/PI. After staining, the apoptotic cell

population was analyzed by Flow Sight Imaging Flow Cytometer (Merck Millipore, Darmstadt, Germany). For each group, 10000 live cells were analyzed.

2.21 Intracellular ROS generation detection. 4T1 cells were seeded into confocal dishes and cultured for 24 h. Fresh DMEM and DIR NPs (20 μg mL^{-1}) were then added into the dishes and cultured overnight. After that, DCHF-DA (20 μM) was added, and the dishes were irradiated under 808 nm laser (0.3 W cm^{-2}) for 0, 30, 60 and 90 s. The confocal images were captured under excitation of 502 nm and the fluorescence of cells was detected on a flow cytometry system.

2.22 *In vitro* examination of immunologic cell death (ICD). To determine ICD induction, surface expression of calreticulin (CRT), high Mobility Group Box 1 (HMGB1) and adenosine triphosphate (ATP) release were studied *in vitro*. 4T1 cells were treated with PBS, PBS + Laser, DIR NPs ([DIR] = 20 μg mL^{-1}), or DIR NPs + Laser (808 nm, 0.3 W cm^{-2}, 5 min). Then the supernatant was collected at 24 h, and the intracellular ATP were determined by ATP Assay Kit. For immunofluorescence staining of surface CRT or HMGB1, 4T1 cells were under different treatments for 24 h, followed by incubation for another 6 h after laser irradiation. Then, the cells were collected and stained with anti-mouse CRT antibody or anti-mouse HMGB1 antibody overnight at 4 °C, followed by incubating with Alexa Fluor 488-labeled Goat Anti-Rabbit IgG secondary antibody for 1 h at room temperature. Then, the cells were stained with DAPI and examined by CLSM.

2.23 *In vitro* direct DC activation. To determine direct DC activation *in vitro*, immature BMDCs were obtained as aforementioned procedures and seeded in 24-well plates (2×10^5 per well), followed by treatment with R848 (5 μg mL^{-1}) and DIR NPs ([DIR] = 20 μg mL^{-1}, [R848] = 5 μg mL^{-1}). After various treatments, the harvested BMDCs were treated with anti-mouse CD16/32 for 20 min at 4 °C to block nonspecific binding and further stained with FITC anti-mouse CD11c, APC anti-mouse CD80, and PE anti-mouse CD86 for 30 min at 4 °C. DC maturation status was evaluated using a Flow Sight Imaging Flow Cytometer (Merck Millipore, Darmstadt, Germany), and was analyzed by FlowJo (Treestar, Ashland, OR, USA).

2.24 *In vitro* DC stimulation transwell experiment. For the activation of DCs induced

by the ICD effect, 4T1 cells were treated with PBS, R848 (5 μg mL^{-1}), DIR NPs ([DIR] = 20 μg mL^{-1}, [R848] = 5 μg mL^{-1}) or DIR NPs + Laser. 4T1 cells were seeded in upper compartment of the transwell system and incubated with DIR NPs for 24 h. Subsequently, the cells were implemented with or without laser irradiation (0.3 W cm^{-2} at 808 nm) for 5 min. Then the immature BMDCs were added in the lower compartment and cocultured for another 24 h. After various treatments, the harvested BMDCs were treated with anti-mouse CD16/32 for 20 min at 4 °C to block nonspecific binding and further stained with FITC anti-mouse CD11c, APC anti-mouse CD80, and PE anti-mouse CD86 for 30 min at 4 °C. DC maturation status was evaluated using a Flow Sight Imaging Flow Cytometer (Merck Millipore, Darmstadt, Germany), and was analyzed by FlowJo (Treestar, Ashland, OR, USA).

2.25 Tumor model establishment. All the mice experiments were carried out in accordance with the guidelines of the Laboratory Animal Center of Jiangsu KeyGEN Biotech Corp., Ltd and approved by the Animal Ethics Committee of Simcere BioTech Corp., Ltd. The License No. is IACUC-404. To establish bilateral tumor models, 4T1 cells (1×10^7) suspended in PBS were subcutaneously implanted into the right flank of each mouse as the primary tumors. Seven days later, 4T1 cells (1×10^7) suspended in PBS were subcutaneously injected into the left flanks of each mouse as distant tumors. The volume of tumor was calculated as follows: $V = Dd^2/2$, V represents the volume of tumor, D and d represent maximum and minimum diameter of tumor, respectively.

2.26 *In vivo* NIR-II fluorescence imaging. 4T1 tumor-bearing BALB/c mice (n = 3) were injected intravenously with DIR NPs (100 μL, 400 μg mL^{-1}). The NIR-II fluorescence images were captured at designated time points (0, 1, 2, 4, 8, 12, 24, 36, and 48 h) post-injection. Fluorescence images were acquired under 808 nm laser excitation with a 980 nm long pass filter, and the exposure time was set as 50 ms. For the ex vivo biodistribution study, mice were sacrificed at t = 48 h post-injection, and the major organs including tumor were collected. The NIR-II fluorescence images of these organs were acquired and the fluorescence intensities were quantified to evaluate the biodistribution of DIR NPs.

2.27 *In vivo* anticancer study. 4T1 tumor-bearing BALB/c mice were randomly

divided into five treatment groups (n = 5), including: PBS; PBS + Laser; R848; DIR NPs, DIR NPs + Laser. Nine days after primary implantation, the mice were administered intravenously with PBS, R848 (5 mg kg^{-1}), or DIR NPs ([DIR] = 2 mg kg^{-1}, [R848] = 5 mg kg^{-1}). At 24 h post injection, tumors on mice from PBS + Laser and DIR NPs + Laser groups were conducted with 808 nm laser at 0.3 W cm^{-2} for 10 min. Afterwards, tumor sizes of primary and distant tumor and body weights of mice were monitored every three days for 21 days. After 21 days of treatments, the mice in each group were euthanized, and both the primary and distant tumors were extracted to weigh. Meanwhile, bilateral tumors were collected for hematoxylin and eosin (H&E) staining.

2.28 Anti-metastasis efficacy evaluation. For the anti-metastasis efficacy evaluation, after 21 days of treatment, lungs of mice in each group (n = 5) were extracted and soaked in Bouin's solution before counting pulmonary metastases and H&E staining.

2.29 Evaluation of intratumoral ICD and cell apoptosis. On the third day following laser irradiation treatment, the primary tumor tissues were extracted and subjected to caspase-3, calreticulin, and HMGB1 immunofluorescence. Then, the secretion levels of ATP and HMGB1 was measured by ATP assay kit and HMGB1 ELISA kit according to the manufacturer's indications, respectively.

2.30 *In vivo* evaluation of DC maturation. 4T1 tumor-bearing BALB/c mice were randomly divided into five treatment groups (n = 5), including: PBS; PBS + Laser; R848; DIR NPs; DIR NPs + Laser. The mice in each group were systemically administrated with PBS, R848 (5 mg kg^{-1}), or DIR NPs ([DIR] = 2 mg kg^{-1}, [R848] = 5 mg kg^{-1}) through tail-vein injection. At t = 24 h, the primary tumors of mice were exposed under 808 nm laser irradiation (0.3 W cm^{-2}) for 10 min. After 3 days of treatment, the mice in each group were euthanized to extract the primary tumors and tumor-draining lymph nodes. The tumor-draining lymph nodes were transferred to a dish and teased into single cells by pressing with the plunger to obtain single cell suspensions. The tumor tissues were harvested and minced into small pieces, followed by digestion in RPMI 1640 medium containing 1 mg mL^{-1} collagenase I, 1 mg mL^{-1} collagenase IV, and 0.1 mg mL^{-1} hyaluronidase at 37 °C for 1 h, then prepared into

single cell suspensions. Before surface staining, the harvested cells were treated with anti-mouse CD16/32 for 20 min on ice to block nonspecific binding, followed by staining with fluorophore-conjugated anti-CD11c, anti-CD80 and anti-CD86 antibodies according to the manufacture protocols. DC maturation status was evaluated using a Flow Sight Imaging Flow Cytometer (Merck Millipore, Darmstadt, Germany), and was analyzed by FlowJo (Treestar, Ashland, OR, USA).

2.31 *In vivo* evaluation of intratumoral T cell population. After 10 days of treatment, 4T1 tumor-bearing BALB/c mice (n = 5) were euthanized to extract primary tumors and tumor-draining lymph nodes. The collected primary tumors and tumor-draining lymph nodes were homogenized into single cell suspension in PBS solution followed by above procedures, respectively. Before surface staining, the harvested cells were treated with anti-mouse CD16/32 for 20 min on ice to block nonspecific binding. The cells were then stained with fluorophore-conjugated anti-CD45, anti-CD3, anti-CD4, anti-CD8, anti-mouse CD44, and anti-mouse CD62L antibodies and analyzed using a Flow Sight Imaging Flow Cytometer (Merck Millipore, Darmstadt, Germany), and was analyzed by FlowJo (Treestar, Ashland, OR, USA).

2.32 Evaluation of serum cytokine levels. Blood samples were collected from 4T1 tumor-bearing BALB/c mice (n = 5) after 3 days of different treatments. The serum levels of IL-6, TNF-α, and IFN-γ were measured using corresponding ELISA kit according to the manufacture protocols.

2.33 *In vivo* toxicity and histological analysis. At the end of *in vivo* therapeutic study, the major organs (heart, liver, spleen, kidney, and intestine) from 4T1 tumor-bearing mice in each group were collected for H&E staining. Liver function was evaluated by determining the serum levels of aspartate aminotransferase (AST) and alanine aminotransferase (ALT). Kidney function was determined by measuring the serum blood urea nitrogen (BUN) and creatinine (CREA) levels.

2.34 Data analysis. Intensities of NIR-II fluorescence images were calculated by region of interest (ROI) analysis using *in vivo* NIR-II fluorescence imaging system. Experimental results were expressed as mean ± SD unless otherwise mentioned. Statistical differences of experimental results were conducted by one-way analysis of

variance (ANOVA) with a Tukey post-hoc test and two-tailed Student's t-test using GraphPad Prism version 8.0.2 (GraphPad Software, Inc., CA, USA). For statistical analysis, **p* < 0.05, ***p* < 0.01, ****p* < 0.001 were regarded as statistically significant.

3. References

[1]. R. C. Benson, H. A. Kues, *J. Chem. Eng. Data* **1977**, *22*, 379.

[2]. L. Lu, B. Li, S. Ding, Y. Fan, S. Wang, C. Sun, M. Zhao, C.-X. Zhao, F. Zhang, *Nat. Commun.* **2020**, *11*, 4192.
